# A review of gallium phosphide nanophotonics towards omnipotent nonlinear devices

**DOI:** 10.1515/nanoph-2024-0172

**Published:** 2024-07-12

**Authors:** Yifan Wang, Ziyu Pan, Yongxian Yan, Yatao Yang, Wenhua Zhao, Ning Ding, Xingyu Tang, Pengzhuo Wu, Qiancheng Zhao, Yi Li

**Affiliations:** School of Microelectronics, 255310 Southern University of Science and Technology, Shenzhen, China; Department of Applied Physics, The Hong Kong Polytechnic University, Hong Kong, China; School of Mechatronics Engineering, Harbin Institute of Technology, Harbin, China; State Key Lab of Advanced Optical Communication Systems and Networks, Department of Electronic Engineering, Shanghai Jiao Tong University, Shanghai, 200240, China

**Keywords:** gallium phosphide, nonlinear optics, optical devices, nano-optics, integrated photonics

## Abstract

Gallium phosphide (GaP) has been increasingly prioritized, fueled by the enormous demands in visible light applications such as biomedical and quantum technologies. GaP has garnered tremendous attention in nanophotonics thanks to its high refractive index, indirect bandgap width of 2.26 eV, lattice perfectly matched with silicon, and omnipotent and competitive nonlinear optical properties. Herein, we review the progress and application of GaP in nanoscale devices over the past two decades. The material properties of bulk GaP are first listed, followed by a summary of the methodologies for fabricating nanoscale devices and related integration techniques. Then, we digest the operational mechanisms across different GaP-based devices on their optical linear responses. Following this, we categorize the GaP nonlinear optical effects into multiple aspects including second-harmonic generation, four-wave mixing, Kerr optical frequency combs, etc. Ultimately, we present a perspective on GaP nanophotonics in the context of coexisting and competing modes of various nonlinear effects. We believe that a comprehensive overview of unique GaP will propel these nanophotonic devices toward a mature state, underpinning foundational understanding and leveraging practical innovations.

## 1 Introduction

Following the success of silicon and germanium devices, compound devices based on the III–V group have been forming a wave toward miniaturization and integration, advanced over various applications and scenarios. Group V primarily includes arsenides, phosphides, and nitrides, functioning over the entire visible and near-infrared (NIR) regime. Traditionally, phosphides receive less attention than arsenides and nitrides, with the only exception being the direct-bandgap indium phosphide (InP) utilized in fiber optical communications. However, a burgeoning interest in gallium phosphide (GaP) photonic devices has emerged recently, especially in nanophotonics.

GaP has been favored in nanophotonics among III/V and other competitors for the following four reasons. (1) GaP has a refractive index above 3.0 over visible and NIR wavelengths, capable of tightly confining light at the interfaces; (2) normal cubic crystalline GaP has an indirect bandgap at 2.26 eV, allowing high transmittance except short wavelengths beyond blue lights; and (3) noncentrosymmetric GaP holds a relatively high second-order nonlinearity of *d*_14_ = 60 pm/V [8] as well as considerable third-order a Kerr nonlinearity 200 times larger than that of silicon nitride (Si_3_N_4_), which opens up the opportunities for odd- and even-order harmonic generations and optical modulation. These properties allow it to be used to create efficient optical modulators and frequency converters. In addition, the lattice constant of GaP (5.451 Å) is intrinsically very close to that of silicon (5.431 Å), advancing its usage to integrate with silicon photonic systems compared to other III–V materials [9]. Other III–V materials, such as GaAs (lattice constant 5.635 Å) and InP (lattice constant 5.869 Å), typically experience lattice mismatch when directly integrated with silicon [10]. Meanwhile, GaP has a lower production cost due to affordable raw materials compared to indium and the handling issues of arsenic [11]. Moreover, GaP possesses good chemical and thermal stability, rendering it a longer operational life and reliability in practical applications. Nevertheless, the GaP devices should be designed and used in suitable conditions to avoid degradation mechanisms like oxidation and power injury. In order to protect the GaP surface from oxidation, Standing et al. [12] created an electrochemically generated oxide passivation layer on the surface of GaP nanowires and Cheng et al. [13] deposited a 2 µm thick silicon dioxide layer on the surface of their designed GaP waveguide devices. Meanwhile, Ye et al. specially studied the intensity dependence of anisotropic third-order optical nonlinearity approaching the damage threshold [14].

**Figure 1:**
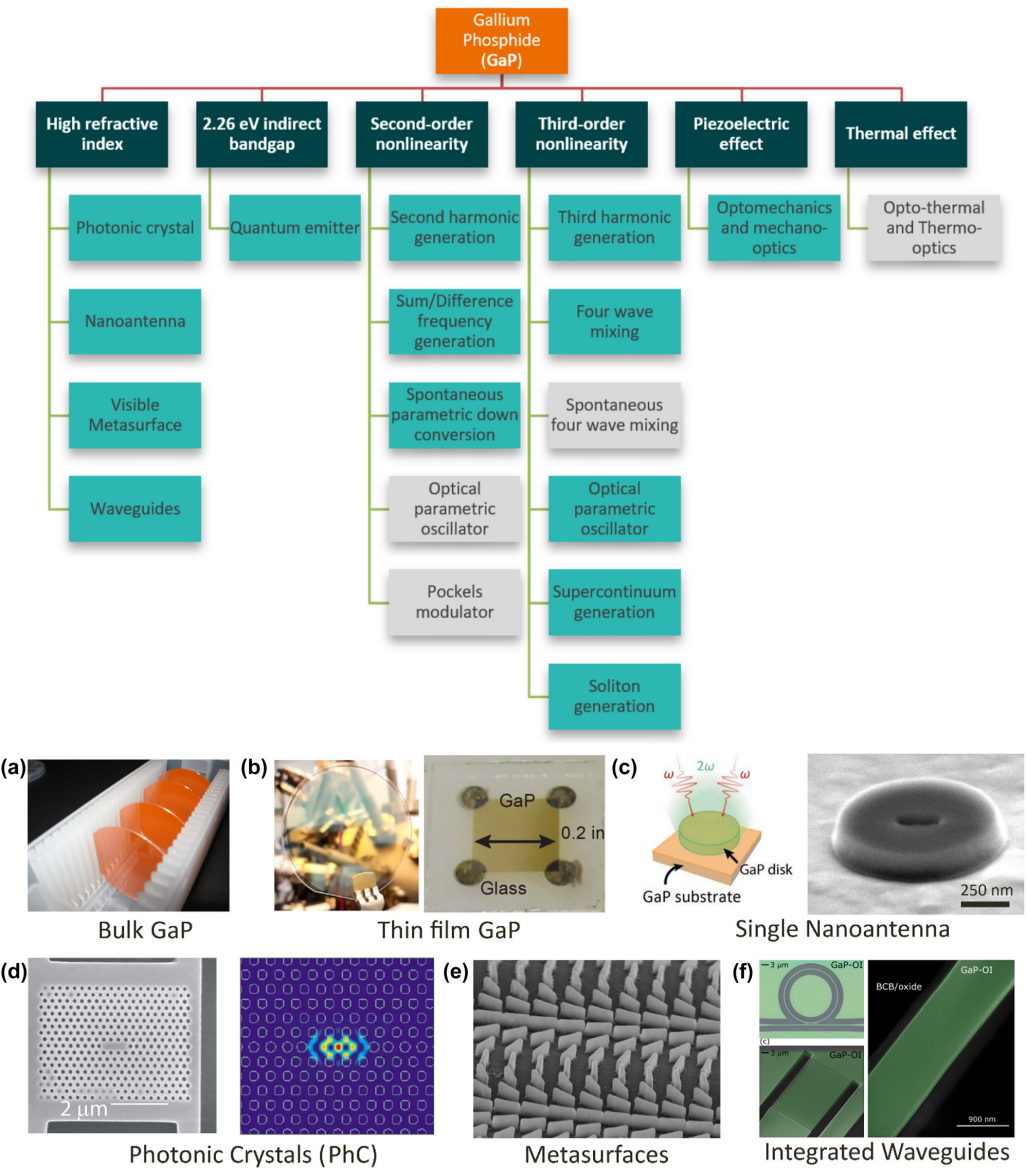
Overview of GaP-based nanodevices. Orange, dark green, and cyan tags indicate material, key properties, and functional devices, respectively. Gray tags indicate the plausible but yet-demonstrated functions. Sample devices for different structures: (a) bulk GaP, (b) thin film GaP, (c) single nanoantenna, (d) photonic crystals (PhCs), (e) metasurface, and (f) integrated waveguides. (a) Reproduced with permission [1]. (b) Reproduced with permission [2]. Copyright 2017, Nature Publishing Group [3]. Copyright 2022, American Chemical Society (c) Reproduced with permission [4]. Copyright 2017, American Chemical Society. (d) Reproduced with permission [5]. Copyright 2008, American Institute of Physics. (e) Reproduced with permission [6]. Copyright 2020, Nature Publishing Group. (f) Reproduced with permission [7]. Copyright 2022, Optical Society of America.

This review summarizes the most recent progress on GaP-based nanodevices, including both passive and active devices demonstrated in the past two decades. A summary of related GaP material properties is presented in Section 2. The fabrication processes, and various device types, such as thin films, single antennas, metasurface-based devices, and integrated waveguides, are discussed in Section 3. The functional devices, including linear and nonlinear optical devices, emitters, photodetectors, and optomechanical devices, are presented in Section 4. Finally, Section 5 concludes this review work with the future outlooks for GaP-based integrated photonics. The overall content of this review is illustrated in Figure 1, where the dark green, orange, and green colored tags represent the material, physical properties, and functional devices, respectively.

## 2 Material properties

### 2.1 Optical properties

Four typical refractive indices of GaP are displayed in Table 1 to shine some light for applications involving wave guiding and photon manipulation. This property is advantageous in creating efficient photonic components like waveguides and lenses. According to Miller’s empirical rule, the higher linear refractive indices are, the higher nonlinear refractive indices exhibit. GaP has a *n*_2_ value goes to 1.13 × 10^−17^ m^2^/W, which is much higher than conventional oxides. As a noncentrosymmetric crystalline material, GaP also exhibits second-order nonlinear susceptibility of *d*_14_ = 60 pm/V [8], making it useful in frequency-doubling applications as well as potentially in Pockel’s effect.

**Table 1:** Material property of GaP for nanodevices.

Property category	Material property	Typical values/characteristics	State-of-the-art	Year	Reference
Optical	Refractive index	2.964 (10 µm), 3.209 (775 nm), 3.590 (500 nm), 5.05 (354 nm)		2016	[21]
Optical	Waveguide loss	1.2 dB/cm (1,550 nm) [16]	0.5 dB/cm	2021	(740 nm) [23]
		6 dB/cm (1,550 nm) [22]	0.85 dB/cm	2023	(1,550 nm) [24]
Optical	Bandgap	2.26 eV (indirect, 300 K)		1996	[17]
Electrical & optical	Nonlinear index		1.13 × 10^−17^ m^2^/W	2020	[68]
Electrical & optical	Electro-optic coefficient	−0.97 × 10^−12^ m/V (623.8 nm)		1968	[25]
Electrical & optical	Second-order nonlinear susceptibility		*d*_14_ = 60 pm/V	2013	[8]
Electrical & mechanical	Piezoelectric coefficient	−0.1 C m^−2^		1996	[17]
Mechanical	Poisson’s ratio	*σ*_o_ = 0.31(300 K, [100])		1990	[26]
Mechanical	Bulk modulus	*B*_s_ = 8.82 × 10^11^ dyn/cm^2^		1990	[26]
	Shear modulus	*C*′ = 3.92 × 10^11^ dyn/cm^2^	
	Young’s modulus	*Y*_o_ = 10.3 × 10^11^ dyn/cm^2^ (300 K, [100])	
Mechanical & optical	Photo-elastic constant	−0.074	−0.082		[27]
Thermal	Thermal conductivity	1.1 W/(cm K) (300 K)		1996	[17]
Thermal & optical	Thermal-optic coefficient	3.4 × 10^−5^ K^−1^		2022	[28]
Thermal & mechanical	Thermal expansion coefficient	5.3 × 10^−6^ K^−1^		2016	[21]

**Figure 2:**
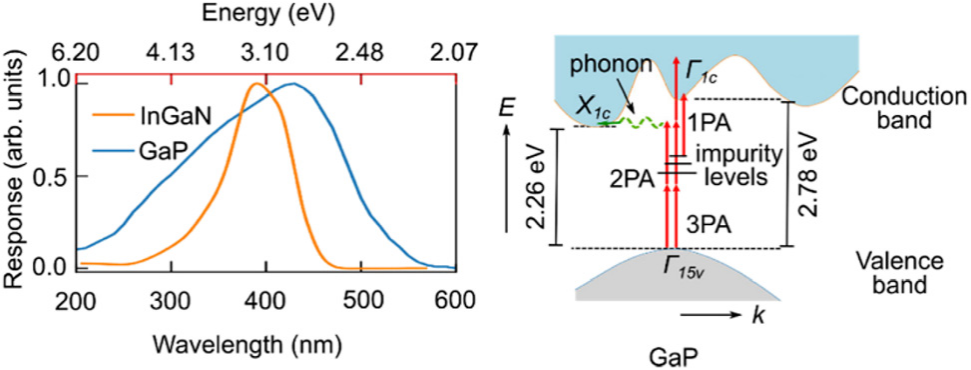
Spectral response and electronic structures of GaP. The indirect transition promotes electrons in the valence band at Γ valley (referred to as Γ_15v_ point) to the conduction band at X valley (referred to as X_1c_ point). This transition requires two-photon absorption (2PA). The lowest direct transition at 2.78 eV promotes electrons from Γ_15v_ to Γ_1c_ with the absorption of three photons (3PA). Electrons from the impurities localized close to the band edge can be promoted to the conduction band by the absorption of a single photon (1PA). Reproduced with permission [15]. Copyright 2023, American Chemical Society.

GaP is an indirect bandgap semiconductor with a bandgap energy of about 2.26 eV at room temperature, shown in Figure 2. This means that photons are not readily emitted when electrons transition from the conduction band to the valence band, which affects their efficiency in light-emitting applications. Selecting nonlinear optical materials requires balancing key parameters summarized in Table 2. To this end, GaP excels with high refractive indices, significant second-order polarizability, and a 2.26 eV bandgap, which secures a minimal two-photon absorption above 1,100 nm for optical transparency. The nature of minimal linear and nonlinear losses not only cover almost all optical communication bands (1,260 nm–1,675 nm) but also enable potential second-/third-order optical modulations in this regime. The GaP can be used in LEDs, but it generally emits light in the red-to-green spectrum. When doped with nitrogen (N), it can produce green light. It has good light absorption properties in the ultraviolet (UV) to visible range, making it useful in photodetector applications.

**Table 2:** Nonlinear optical materials and their optical coefficients at 1,550 nm unless stated otherwise.^a^

Material	Optical refractive index	Bandgap (eV)	Second-order nonlinear coefficient (pm/V)	Nonlinear refractive index (10^−18^ m^2^/W)	Year	Ref
Si	3.5	1.1	–	4	2003	[29]
Al_0.17_Ga_0.83_As	3.3	1.63	210	26	2016	[30]
GaP	3.05	2.26	60	11.3	2020	[16]
					2023	[8]
Diamond	2.4	5.5	–	0.082	2014	[31]
GaN	2.3	3.4	16	3.4	2011	[32]
					2018	[33]
Si_3_N_4_	2	5	–	0.25	2017	[34]
SiO_2_	1.45	9	–	0.022	2012	[35]
Doped silica	1.44	0	–	0.022	2008	[36]
AlN	2.12	6.2	4.7	0.23	2013	[37]
					2012	[38]
4H-SiC	2.6	3.26	11.7 @ 1064 nm	0.6	2023	[39]
					2019	[40]
Lithium niobate	2.2	3.78	−27 @ 1064 nm	0.18	2021	[41]
Lithium tantalate	*n*_e_ = 2.123	3.93	−21 @ 1064 nm	0.3 ± 0.06 @ e-wave 800 nm	2024	[42]
	*n*_o_ = 2.117			0.17 ± 0.03 @ o-wave 800 nm	2003	[43]
GaAs	3.4	1.42	238 @ 1533 nm	20	1997	[44]
					2007	[45]
As_2_S_3_	2.38	2.4	–	2.92	2007	[46]

^a^Wavelengths are set at 1,550 nm unless stated otherwise.

**Figure 3:**
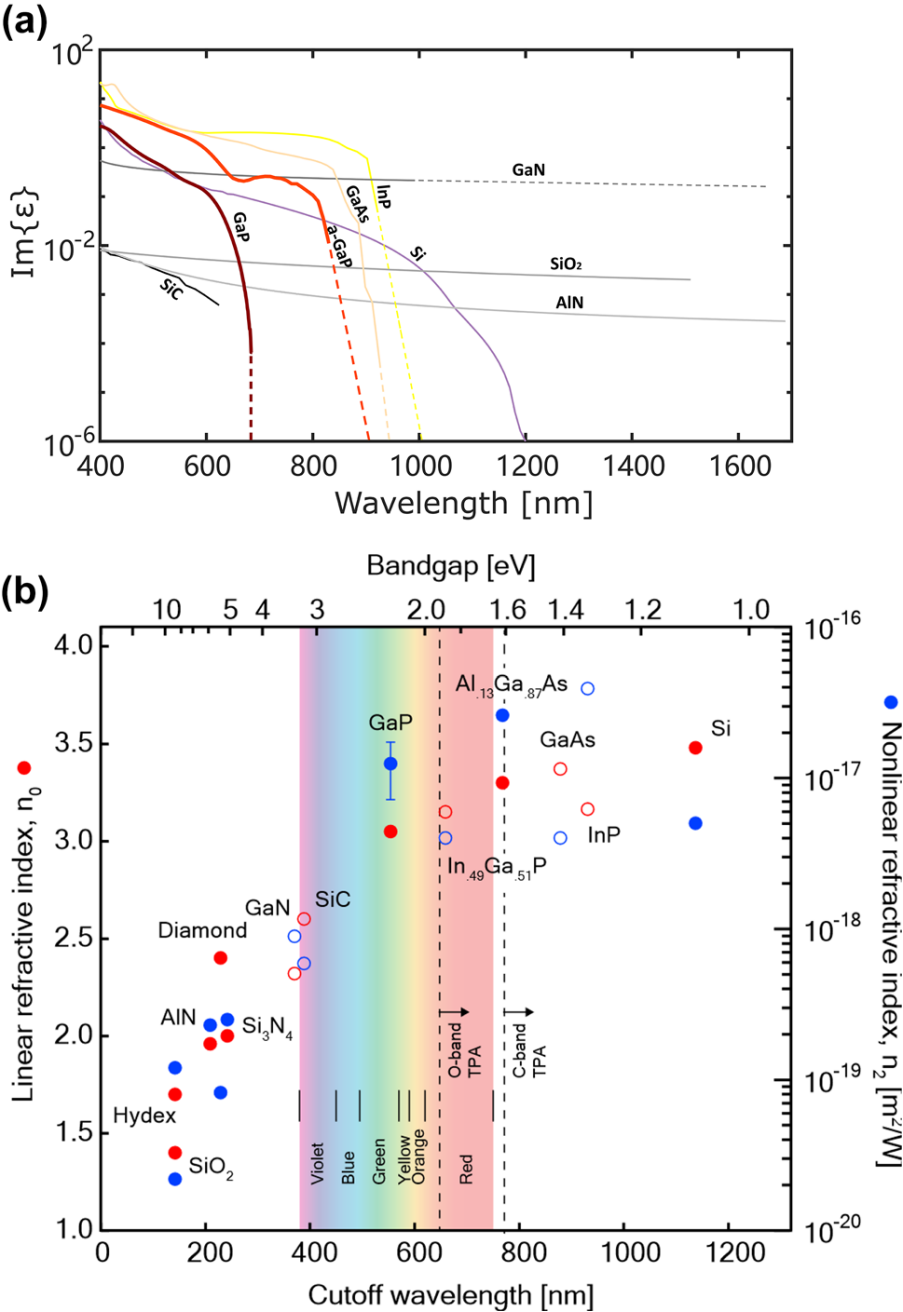
Optical losses (a), linear and nonlinear indices (b) as a function of wavelengths. (b) Reproduced with permission [16]. Copyright 2019, Nature Research.

We have compared the optical loss and second-order nonlinearity of GaP with other materials in Figure 3. On one hand, stemmed from the band structure of GaP shown in Figure 2, the indirect bandgap at 2.26 eV (*X*_1c_) set one bar at 548.6 nm, while its direct bandgap at 2.78 eV (Γ_1c_) set the other at 446.0 nm. Thus, the loss for the wavelength shorter than 446.0 nm is reasonably high due to absorption, while the loss for the wavelength longer than 548.6 nm drastically declines in Figure 3(a). The optical loss of crystalline gallium phosphide (c-GaP) significantly outperforms amorphous gallium phosphide (a-GaP) in terms of optical loss, primarily due to its ordered crystal structure and lower defect density. On the other hand, the nonlinear refractive index of GaP (1.13 × 10^−17^ m^2^/W) is nearly two orders of magnitude higher than that of other commonly used integrated optical materials, such as silicon carbide (SiC) and aluminum nitride (AlN). These two advantages position GaP a highly promising candidate material for use in photonic integrated circuits.

### 2.2 Piezoelectric properties

Piezoelectric constant e_14_ of GaP equals to −0.1 C m^−2^ [17]. Conventionally, GaP is not recognized as a piezoelectric material in its standard form. However, the recent progresses of GaP at the nanoscale in turn exhibit significant piezoelectric response, making it extremely well suited in a variety of novel applications for optomechanical quantum systems.

### 2.3 Mechanical and acoustic properties

How a material deforms under stress can be reflected by bulk modulus, shear modulus, and Young’s modulus. Bulk modulus (*B*_s_) measures how a material responds to uniform compression. A bulk modulus as high as 8.82 × 10^11^ dyn/cm^2^ indicates that GaP is quite incompressible. Shear modulus (*C*′) is known as the modulus of rigidity, measuring the response to shear stress, which is the force that causes different layers of the material to slide past each other. It quantifies how a material deforms under such a force. A shear modulus of 3.92 × 10^11^ dyn/cm^2^ means GaP is quite rigid and less prone to shearing. Young’s modulus (*Y*_o_) refers to the modulus of elasticity in tension, measuring a material’s stiffness. It describes how a material stretches under longitudinal stress. A Young’s modulus of 10.3 × 10^11^ dyn/cm^2^ indicates a stiff material.

Meanwhile, Poisson’s ratio is also a measure of the elasticity of a material, named after the French mathematician and physicist Siméon Poisson. It describes the relationship between longitudinal strain and lateral strain in a material when it is stretched or compressed. Specifically, Poisson’s ratio (*σ*_o_) is defined as the negative ratio of the transverse (lateral) strain to the axial (longitudinal) strain. When a material is stretched or compressed, longitudinal strain (deformation) is present in the direction of the applied force. Transverse strain is perpendicular to the direction of the applied force. Given that the value of Poisson’s ratio is typically between −1.0 and 0.5 for most materials, *σ*_o_ of 0.31 for GaP exhibits very limited volume change when deformed.

Phonons are quasiparticles representing quantized vibrational energy in the crystal lattice of a solid. The frequencies of these vibrations vary based on the material’s atomic structure and the nature of the atomic bonds. Four phonon frequencies at 4.2 K are remarkably cited here for both acoustic phonons and optical phonons on longitudinal (parallel to the wave vector) or transverse (perpendicular to the wave vector) directions (LA, TA, LO, and TO). On the one hand, acoustic phonons involve the coherent motion of atoms in the same direction, which usually have lower frequencies and are important in thermal conductivity. On the other hand, optical phonons, which involve the motion of atoms in opposite directions within the basis of the unit cell, typically have higher frequencies, specifically for GaP, 11.3 THz for *ν*_LO_, 10.96 THz for *ν*_TO_, 7.65 THz for *ν*_LA_, and 3.16 THz for *ν*_TA_. They can also be estimated using theoretical methods based on lattice dynamics and quantum mechanics.

### 2.4 Thermal properties

The GaP has a high melting point of around 1,457 °C, up to which GaP is thermally stable, rendering GaP suitable for high-temperature applications and processing. At room temperature (300 K), GaP has a moderate thermal conductivity, typically around 110 W/(mK) [17], which is comparable to that of many other semiconductors, such as Si (130 W/(m K) [18]), but lower than materials like copper or diamond.

The coefficient of thermal expansion (CTE) for GaP is approximately 5.3 × 10^−6^ K^−1^, nearly ten times larger than that of SiO_2_ (0.56 × 10^−6^ K^−1^ [19]). Thus, it is important to consider the CTE mismatches in heterogeneous GaP photonic devices.

The specific heat capacity of GaP is approximately 0.43 J/g °C [20], which represents the amount of heat required to raise the temperature of a unit mass of material by one degree Celsius. This parameter is useful to determine the thermodynamics of the GaP photonic devices.

### 2.5 Bandgap properties

GaP is transparent to infrared light, which is beneficial in applications where infrared transmission is required. It has good light absorption properties in the ultraviolet (UV) to visible range, making it useful in photodetector applications. As an indirect bandgap semiconductor with a bandgap energy of about 2.26 eV, GaP is not an efficient light-emitting material. It could be used in light-emitting diodes (LEDs), but it generally emits light in the red to green spectrum. When doped with nitrogen (N), it can produce green light.

### 2.6 InGaP and AlGaP

In contrast, GaP’s alloys, such as indium gallium phosphide (InGaP), and aluminum gallium phosphide (AlGaP), can have direct tunable bandgaps depending on the material compositions. The ternary semiconductor material InGaP has a bandgap energy spanning from 1.9 eV (with high indium content) to about 1.35 eV (lower indium content). Compared to GaP (2.26 eV) and GaAs (1.423 eV), InGaP is more efficient for high-efficiency solar cells, LEDs, and laser diodes, particularly valuable in multi-junction solar cells where layers with different bandgaps are needed to absorb different wavelengths of light more efficiently.

AlGaP also has a direct bandgap energy tuned by varying the aluminum-to-gallium ratio from about 2.26 eV (similar to pure GaP) up to approximately 2.4 eV. The existence of aluminum atoms manifests itself as a great sacrificial layer for the fluoride-based wet etching process.

## 3 Fabrication approaches and device architectures

### 3.1 Fabrication

#### 3.1.1 Bulk GaP

GaP single crystals are commonly produced in high-pressure synthesis furnaces. The process begins with the preparation of high-purity gallium and phosphorus, the primary constituents of GaP. The initial step involves the synthesis of GaP polycrystals using a directional solidification process in a high-pressure furnace. These polycrystals are then appropriately treated and loaded into a high-pressure single-crystal furnace for crystal pulling. During the crystal pulling process, a seed crystal of GaP is immersed into the molten material. This seed crystal is then gradually withdrawn from the melt while being rotated. This controlled movement and rotation help promote the growth of a single crystal, ensuring uniformity and minimizing defects. An important aspect of this process is the Liquid Encapsulation Czochralski (LEC) method. In LEC, the molten GaP is covered with a layer of inert liquid, such as boron trioxide (B_2_O_3_). This encapsulation serves a crucial role in preventing the evaporation of phosphorus, which is volatile at high temperatures. By maintaining the integrity of the phosphorus content in the melt, the quality and consistency of the GaP crystal are enhanced. Finally, the crystal is sliced into thin wafers for further processing in semiconductor fabrication.

#### 3.1.2 Thin film GaP

**Figure 4:**
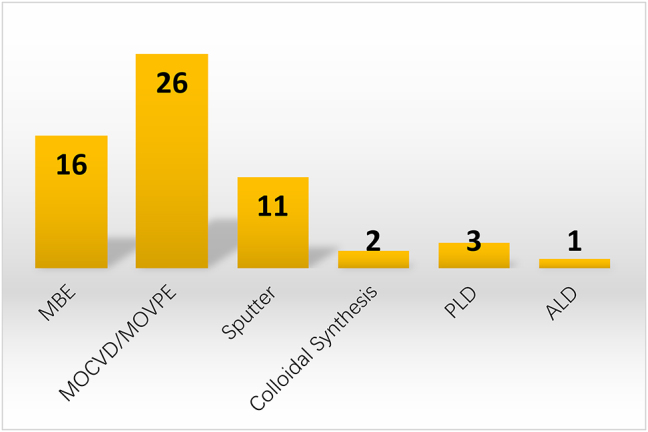
Deposition methods for GaP thin films.

High-quality GaP films with tailorable properties are desired in nanophotonic devices. GaP thin film can be fabricated by variant methods and qualities, including Molecular Beam Epitaxy (MBE), Metal–Organic Chemical Vapor Deposition or Metal–Organic Vapor Phase Epitaxy (MOCVD/MOVPE), sputtering, colloidal synthesis, pulsed laser deposition (PLD), as well as atomic layer deposition (ALD). Figure 4 shows the statistics of the GaP film growth methods in the publications.

MBE is particularly useful for GaP growth because it produces very pure and high-quality crystalline layers with precise control over composition and thickness. MBE involves the deposition of constituent atomic or molecular beams onto a substrate under ultra-high vacuum conditions. This environment allows for a well-controlled growth process, layer by layer. In the case of GaP, gallium (Ga) and phosphorus (P) crackers are placed in separate effusion cells within the MBE chamber. These cells are heated to precisely controlled temperatures to produce beams of gallium and phosphorus atoms. A suitable substrate, often a single-crystal wafer such as Si or GaP itself, is prepared and placed in the chamber. The substrate is heated to an appropriate temperature for GaP growth. Ga and P atoms are directed toward the substrate, condensing and reacting to form GaP. The growth rate is controlled by the temperatures of the effusion cells and the substrate, as well as the beam fluxes. Techniques like Reflection High-Energy Electron Diffraction (RHEED) are employed to monitor the crystal structure during growth in real time. On the other hand, MBE is a complex and costly process requiring sophisticated equipment and ultra-pure materials, which is generally more suited for research and development or specialized applications rather than mass production.

MOCVD/MOVPE is widely used to grow III–V semiconductors like GaAs, InP, GaN, etc. It’s particularly valuable in producing optoelectronic devices, lasers, and LEDs, where precise material composition and crystalline structure control is crucial. MOCVD/MOVPE is a process that uses metal–organic precursors (like trimethylgallium for gallium and phosphine for phosphorus). These gases are transported into a reaction chamber with a carrier gas (often hydrogen). In the chamber, the substrate (like a Si, GaP, or GaAs wafer) is heated to a high temperature. The metal–organic precursors react on the surface of the heated substrate, leading to the deposition of a thin layer of GaP. By adjusting the flow rates of the precursor gases, the temperature, and the pressure in the chamber, it’s possible to control the thickness and composition of the GaP layer with high precision. This method offers advantages such as uniform deposition over large areas, precise control over the stoichiometry and thickness of the layers, and the ability to grow complex, multilayer structures for advanced semiconductor devices. However, it is very susceptible to the cleanness of the substrate as well as the growth condition. The crystalline thin films deposited via MOCVD is comparable with bulk GaP wafers (53 pm/V) holding a *χ* ^(2)^ of 55 pm/V at 1,050–1,400 nm wavelength range as shown in Table 3. This value exceeds that of most common nonlinear crystals, such as BBO, (D)KDP, or LiNbO_3_, surpassing the latter two by an order of magnitude [8].

**Table 3:** Second-order nonlinear susceptibility for several nonlinear materials [8].

Material	Thickness	Wavelength [μm]	*χ* ^(2)^ [pm V^−1^]
GaP	Bulk	1.3	53
c-GaP	400 nm	1.05–1.4	55
MoS_2_	Bulk	1.56	29
GaAs	Bulk	1.55	120
BBO	Bulk	1.064	3.7
LiNbO_3_	Bulk	1.5	20–30
(D)KDP	Bulk	1.064	0.39
Al_1−*x*_SC_*x*_N	500 nm	1.54	60

Atomic Layer Deposition (ALD) is a precise thin film deposition technique used to grow uniform layers of materials with atomic-level control. ALD involves self-limiting surface reactions between precursor molecules and the substrate surface. These reactions occur sequentially, allowing for precise control over film thickness and composition. For GaP deposition, suitable precursors containing gallium and phosphorus are needed. Organometallic compounds like trimethylgallium (TMGa) and tritertiary butyl phosphine (tri-TBP) are specifically used as precursors due to their volatility and reactivity [47]. The ALD process consists of alternating pulses of the gallium precursor and the phosphorus precursor. During each pulse, a monolayer of precursor molecules chemisorbs onto the substrate surface and reacts to form a thin layer of GaP. Between precursor pulses, the chamber is purged with an inert gas (e.g., nitrogen) to remove any unreacted precursor and reaction by-products, ensuring clean and well-defined interfaces between layers. ALD of GaP typically occurs at elevated temperatures (usually between 300 °C and 500 °C) to facilitate precursor reactions and promote film growth. The process is carried out under low-pressure conditions to minimize gas-phase reactions and achieve uniform film growth. The precise control offered by ALD enables the fabrication of high-quality GaP films with tailored properties for specific applications.

Sputtering is a physical vapor deposition (PVD) process where atoms are ejected from a solid target material due to bombardment of the target by energetic particles, usually ions, in a plasma. In the case of GaP sputtering, the target is typically a solid piece of GaP placed in a vacuum chamber. The sputtering chamber is filled with an inert gas, such as argon. When a high voltage is applied, the gas is ionized into plasma. The positively charged ions in the plasma are accelerated toward the negatively charged GaP target, which sometimes could be brittle into pieces. When the argon ions hit the GaP target, they knock off (or “sputter”) atoms from the target. These GaP atoms then travel through the vacuum chamber and deposit onto the substrate, forming a thin film. The substrate, which can be various materials depending on the intended use, is positioned in the chamber opposite the GaP target. The distance and angle between the target and the substrate can affect the uniformity and quality of the film. The properties of the sputtered GaP film, such as its thickness and uniformity, can be controlled by adjusting various parameters like the power applied to the target, the pressure and composition of the sputtering gas, the time duration of sputtering, and the substrate temperature. Sputtering of compound materials like GaP can be challenging because of the difference in sputtering yield between gallium and phosphorus. This can lead to changes in stoichiometry from the target to the deposited film. To address this, techniques like reactive sputtering or cosputtering might be employed. Sputtering is favored in certain applications due to its ability to provide uniform films over large areas and deposit materials on substrates with lower temperatures than other methods like Chemical Vapor Deposition (CVD). However, controlling the stoichiometry and properties of the deposited GaP layer can be more challenging than other methods. The amorphous sputtered thin films show negligible *χ* ^(2)^ due to the lack of noncentrosymmetry, which is in line with our understanding the sputtering process. Counterintuitively, *χ* ^(3)^ of c-GaP film is approximately one third of bulky GaP (3.015 × 10^−20^ m^2^/V^2^), while a-GaP with the exact same thickness exhibits 25.6× enhancement of c-GaP film as shown in Table 4 [8].

**Table 4:** Third-order nonlinear optical susceptibility values for selected materials [8].

	Thickness	Wavelength [μm]	*χ* ^(3)^ [10^−20^ m^2^/V^2^]
GaP	Bulk	1.680	3.015
c-GaP	400 nm	1.680	1.0
a-GaP	400 nm	1.680	25.6
Cuq_2_	166 μm	1.064	1.85
Ge	1.6 μm	1.650	56.5
Si	Bulk	1.650	3.84
ITO	310 nm	1.24	450
MoS_2_	Bulk	1.560	24

PLD is another physical vapor deposition (PVD) technique where a high-energy laser pulse is used to ablate material from a target. The ablated material then deposits onto a substrate placed opposite to the target in a vacuum chamber. When a high-energy laser pulse is directed onto the GaP target, it rapidly heats and ablates material from the target surface. This creates a plasma plume consisting of GaP atoms and ions. The GaP atoms and ions in the plasma plume travel across the vacuum chamber and deposit onto the substrate. The substrate is typically heated to promote adhesion and crystalline growth of the deposited material. The properties of the deposited GaP film can be controlled by adjusting various parameters such as the laser energy, pulse duration, repetition rate, target-substrate distance, substrate temperature, and the background gas pressure in the chamber. In contrast with conventional sputtering, PLD allows for precise control over the stoichiometry of the deposited GaP film. By adjusting the laser parameters and the deposition conditions, it is possible to achieve the desired Ga-to-P ratio in the deposited film. PLD offers advantages such as high deposition rates, stoichiometric control, and the ability to deposit complex multicomponent films. However, challenges include the need for optimization of laser parameters and the potential for substrate damage due to the high-energy laser pulses.

Colloidal synthesis is frequently utilized in preparing GaP nanowires and nanoparticles. It typically starts with selecting of suitable precursors containing gallium and phosphorus. These precursors should react under appropriate conditions to form GaP nanoparticles. GaP nanowires can be synthesized by triethylgallium (TEG) and tris(trimethylsilyl)phosphine (TMSP) precursors (1:1 M ratio) with squalane being the solvent. The product yield reached ∼80 % under the optimized conditions [48]. Functional GaP nanowires can be achieved by adding dopants during the colloidal synthesis process. For p-type shallow dopants, typically Zn is necessary to control the acceptor concentration. Diethylzinc (DEZn) has been proven useful as the source of Zn for this type of synthesis [49].

#### 3.1.3 Bonding

**Figure 5:**
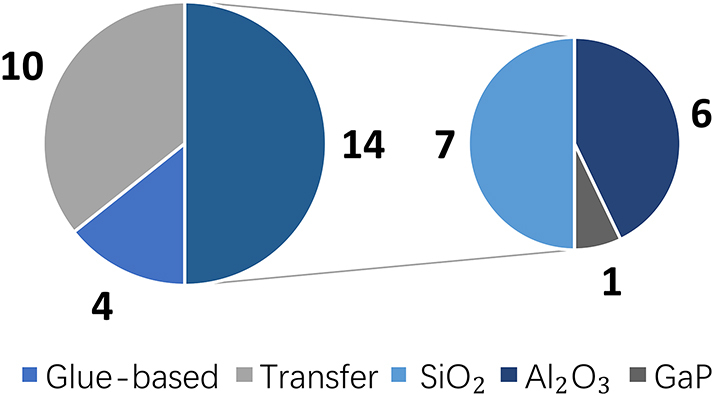
Bonding methods for GaP thin films. Direct bonding (14) includes SiO_2_ (7), Al_2_O_3_ (6), and GaP (1) interface.

Limited by lattice matching requirement, crystalline GaP thin films need to be bonded to other substrates to form heterogeneous structures. The contacting surfaces of the GaP wafers must be meticulously cleaned and polished. This usually involves chemical cleaning to remove contaminants, oxides, or residues. Surface roughness is minimized to ensure a strong bond, particularly GaP Hillock defects [50], where chemical mechanical polishing may become compulsory. Alignment also plays a critical role, which is typically implemented using specialized equipment that can align wafers with high accuracy. Figure 5 shows the statistics of bonding methods for GaP thin films in the publications. A few bonding methods have been reported and will be enumerated below.

Direct wafer bonding requires the cleaned and activated surfaces of the wafers. Natural molecular forces (like van der Waals forces) can create a bond without gluing [51]. The bonding yield could be further improved with the assistance of thin dielectric layers such as SiO_2_ [8], [52], [53], [54], [55], [56], [57] and Al_2_O_3_ [16], [23], [24], [58], [59], [60] Similar to direct wafer bonding, both anodic bonding and fusion bonding involve directly contacting GaP surfaces with other substrates. The difference is that anodic bonding establishes an electrostatic bond by applying heat and high voltages, while fusion bonding needs heating after contact to strengthen the bond.

Adhesive bonding utilizes a thin layer of adhesive (such as epoxy or polymer) between wafers to glue the heterogeneous layers [13], [61], [62]. The critical aspect of this process lies in preventing micro- or nanobubble formation within the adhesive layer. Should these bubbles arise, there exists a risk of them enlarging progressively, potentially leading to the cracking of GaP films.

Micro-transfer printing (*µ*TP) is an innovative technique that allows for the heterogeneous integration of micro and nanostructures made from various materials onto different target substrates. The prefabricated structures are picked with a PDMS stamp and transferred onto the target surface with minimal postprocessing requirements. With *µ*TP it is possible to do mass production of integrated devices with ±0.5 µm 3*σ* alignment accuracy and arrays of devices can be transferred in a single step, to achieve a high throughput [7], [22], [63].

#### 3.1.4 Device patterning

**Figure 6:**
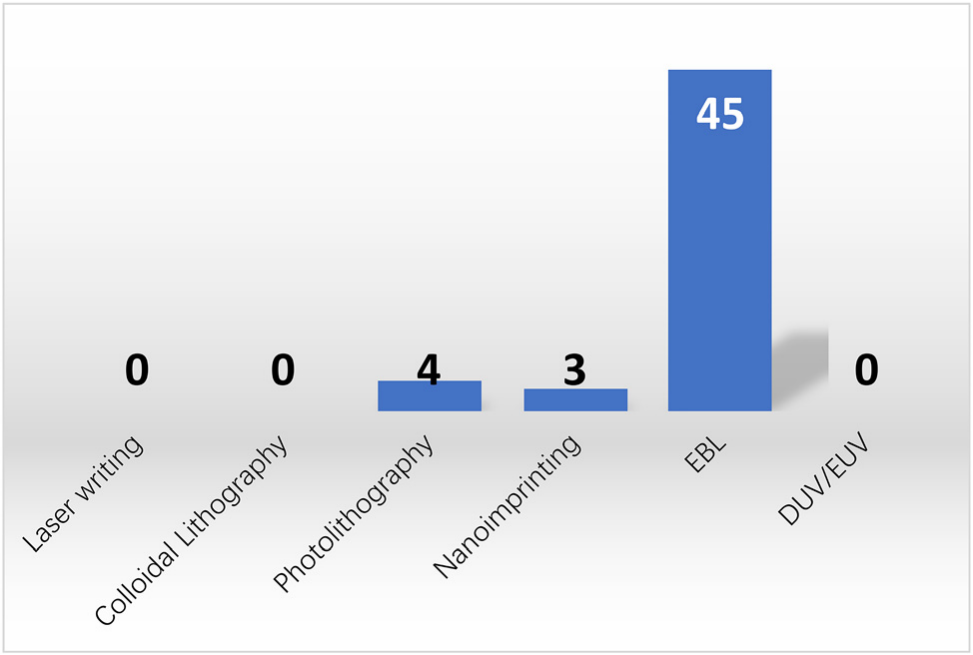
Lithography methods for GaP devices.

Micro/nanopatterns rely on the advanced lithography processes. Electron Beam Lithography (EBL) is a method of patterning nanostructures, often used in nanotechnology and semiconductor fabrication. As shown in Figure 6, 45 out of 52 works utilize the EBL method for patterning, far exceeding the second place being the photolithography method. Photolithography has the advantage of large-scale patterning at the cost of minimum resolution. The resolution constraints could be mitigated by using advanced lithography tools such as deep ultraviolet lithography machines but with higher expenses. Another delicate patterning method is nanoimprinting lithography (NIL). It begins with creating a master mold or template. This master mold, often fabricated by EBL or photolithography, is uniformly pressed into the resist-coated GaP substrate under specific conditions of temperature and pressure. It is worth noting that managing the mechanical and thermal stresses during imprinting, ensuring the fidelity of pattern transfer, and dealing with any potential damage to the GaP substrate becomes essential to ensure the quality and effectiveness of the nanostructures created. Considering scalability, NIL could be a better patterning method than EBL, as the scanning-based latter one is time-consuming, expensive, and restrictive on device sizes [64]. At the moment, neither laser writing, colloidal lithography, nor Deep/Extreme Ultraviolet (DUV/EUV) lithography has been applied for GaP nanophotonic devices.

#### 3.1.5 Etching

The etching of GaP lies at the heart of transferring patterns into the core material. There are two primary methods of etching GaP: wet etching and dry etching.

Wet etching involves using chemical solutions to etch GaP. Common etchants include a mixture of hydrochloric acid (HCl) and hydrogen peroxide (H_2_O_2_), or sulfuric acid (H_2_SO_4_). It is relatively simple and cost-effective, suitable for large-area etching, and does not require sophisticated equipment.

GaP dry etching employs gases or plasmas, including chlorine (Cl_2_), silicon tetrachloride (SiCl_4_), and sulfur hexafluoride (SF_6_). Inductively Coupled Plasma – Reactive Ion Etching (ICP-RIE) is the most widely used dry etching method, as summarized in Table 5. It is worth noting that ICP-RIE is a sensitive process with many parameters, causing day-by-day variations in the etch rate, so calibration of the etch rate is recommended before fabricating actual devices. A successful dry etching on GaP depends not only on the tool parameters but also on the masking materials, which will be elaborated more in the following section.

**Table 5:** Process flow and statistics of GaP devices.

Structure	Deposition	Bonding	Mask	Patterning	Etching	RTA	Annealing	Year	Reference
Bulk								2015	[68]
Bulk								2020	[69]
Bulk (100)								2019	[70]
Bulk (100)								2020	[71]
Film	Sputtering							1970	[72]
Film	Nanoparticle colloidal suspension deposition on a quartz substrate							2010	[73]
Film	RF magnetron sputtering							2011	[74]
Film	GaP/fused silica substrate RF sputtered.							2011	[75]
Film	GaP/(Si (001)/Si(112)) MOCVD	SU-8						2017	[2]
Film	AlGaP(100 nm)/GaP (100) MOCVD		SiO_2_	UV lithography	ICP-RIE (SF_6_/SiCl_4_)			2018	[76]
Film	GaP/Si(500 °C) pulsed laser deposition (PLD)							2021	[77]
Film	GaP(400 nm)/AlGaInP/GaAs MOCVD	Directly bonding by 2 µm SiO_2_ on both sides						2021	[55]
Film	GaP(400 nm)/AlGaInP/GaAs MOCVD	Directly bonding by 2 µm SiO_2_ on both sides						2022	[56]
Film	c-GaP(400 nm)/GaAs MOCVD	Directly bonding by 5 µm SiO_2_ on both sides						2023	[8]
	a-GaP/cover glass (350 °C) RF sputtered								
Film	GaP/AlGaP/GaP epitaxially grown	Micro-transfer printing	PR	UV lithography	ICP-RIE			2023	[22]
Film	GaP (200) (20 nm)/In_0.5_Ga_0.5_P (200)/GaAs ALD							2024	[47]
Metasurface	RF sputtering on silica and sapphire(450 °C and 550 °C)		HSQ	EBL	ICP-RIE	750 °C 5 min		2020	[6]
Metasurface	GaP (400 nm)/AlGaInP/GaAs MOCVD	Directly bonding by 2 µm SiO_2_ on both sides	HSQ (540 nm)	EBL	ICP-RIE (N_2_/Cl_2_)			2020	[53]
Metasurface	GaP (111) (200 nm)/sapphire (0001) MBE		Metal	EBL	ICP-RIE (BCl_3_/Cl_2_)			2021	[78]
Metasurface	a-GaP (100 nm)/ITO/a-SiO_2_(350 °C) RF sputtered		Nanoimprint/EBEAM RESIST	Nanoimprint/EBL	ICP-RIE (Ar 30 sccm/Cl_2_ 10 sccm)			2021	[64]
Metasurface	GaP (100) (400 nm)/AlGaInP/GaAs MOCVD	Directly bonding by 2 µm SiO_2_ on both sides	HSQ (∼200 nm)	EBL	ICP-RIE (N_2_/Cl_2_)			2021	[57]
Metasurface	GaP (210 nm)/Si CVD		ZEP520A	EBL	ICP-RIE (Ar/Cl_2_)			2023	[79]
Metasurface	GaP (400 nm)/AlGaInP/GaAs MOCVD	Directly bonding by 2 µm SiO_2_ on both sides	HSQ (540 nm)	EBL	ICP-RIE (N_2_/Cl_2_)			2023	[80]
Microdisk	GaP (250 nm)/AlGaP (750 nm)/GaP epitaxially grown		ZEP520A	EBL	ICP-RIE (Ar/Cl_2_)			2014	[81]
Microdisk	GaP (250 nm)/AlGaP (750 nm)/GaP epitaxially grown		ZEP520A	EBL	ICP-RIE (Ar/Cl_2_)			2016	[28]
Microdisk	GaP (250 nm)/AlGaP (750 nm)/GaP epitaxially grown		ZEP520A	EBL	ICP-RIE (Ar/Cl_2_)			2022	[82]
Nanoantenna	Bulk GaP (100)		SiO_2_	EBL	ICP-RIE (Ar 30 sccm/Cl_2_ 12 sccm)			2017	[4]
Nanoantenna	Bulk GaP (100)		SiO_2_	EBL	ICP-RIE (Ar 30 sccm/Cl_2_ 12 sccm)			2019	[83]
Nanoantenna	GaP (amorphous)/glass (250 °C) RF sputtered		SiO_2_	EBL	ICP-RIE (Ar 30 sccm/Cl_2_ 10 sccm)			2020	[84]
Nanoantenna	Bulk GaP (100)		SiO_2_	EBL	ICP-RIE (Ar 30 sccm/Cl_2_ 12 sccm)			2021	[85]
Nanoantenna	Bulk GaP (100)		SiO_2_	EBL	ICP-RIE (Ar 30 sccm/Cl_2_ 12 sccm)			2021	[85]
Nanoantenna	GaP (111)/Al_2_O_3_ (0001) MBE		Au	EBL	ICP-RIE (BCl_3_/Cl_2_)			2022	[86]
Nanoantenna	GaP (50 nm)/glass (350 °C) sputter		Au	EBL	ICP-RIE			2023	[87]
Nanoantenna	Bulk GaP (100)		SiO_2_	EBL	ICP-RIE (Ar 30 sccm/Cl_2_ 12 sccm)			2024	[88]
Nanodisk	Bulk GaP (100)		SiO_2_	EBL	ICP-RIE (Ar 30 sccm/Cl_2_ 12 sccm)			2019	[89]
Nanodisk	GaP (400 nm)/GaAs MOCVD	Directly bonding by 5 µm SiO_2_ on both sides	HSQ	EBL	ICP-RIE (N_2_/Cl_2_)			2020	[54]
Nanodisk	GaP/SiO_2_ cover glass (350 °C) RF sputtered		EBEAM RESIST	EBL	ICP-RIE (Ar/Cl_2_)			2021	[90]
Nanoparticles	Combination of mechanical milling and a pulsed laser melting in solution process							2023	[91]
Nanopillar	Bulk GaP (100)		Silica colloidal particles		ICP-RIE (Ar/Cl_2_)			2012	[92]
Nanopillar	Bulk GaP (100)		SiO_2_	EBL	ICP-RIE (H_2_ 10 sccm/Cl_2_ 14 sccm/CH_4_ 5.5 sccm)			2014	[93]
Nanowire	GaP (111)/crystalline silicon PLV (pulsed laser vaporization) VLS							2003	[94]
Nanowire	Surfactant-free solution-liquid-solid (SLS) synthetic method							2012	[49]
Nanowire	Al_0.4_Ga_0.6_P/GaP/GaP (111)_B_ MOVPE VLS			Nanoimprint/EBL				2013	[95]
Nanowire	GaP/GaP (111)_B_ MOVPE VLS			Nanoimprint				2015	[12]
Nanowire	GaP/Si(111) MBE							2020	[96]
Nanowire	GaP/glass MBE							2020	[86]
Nanowire	GaP/GaPAs/GaP/Si (111) MBE							2021	[97]
Nanowire waveguide	GaP/(SiO_x_/Si (111)) MBE self-catalytic VLS							2023	[98]
Optomechanical resonator	GaP (200 nm) (110)/Al_0.64_Ga_0.36_P (1 µm)/GaP epitaxially grown							2019	[99]
Optomechanical resonator	GaP (200 nm) (110)/Al_0.64_Ga_0.36_P (1 µm)/GaP epitaxially grown		AR-P 6200-13	EBL	ICP-RIE (N_2_/Cl_2_/BCl_3_)			2022	[100]
PhC	GaP (140 nm)/AlGaP (1 µm)/GaP (100) MBE		ZEP520	EBL	ICP-RIE			2008	[5]
PhC	GaP (160 nm)/AlGaP (1 µm)/GaP (100) MBE		ZEP520	EBL	ICP-RIE			2010	[101]
PhC	GaP (70 nm)/Si (100) MOVPE			EBL				2010	[102]
PhC	GaP (230 nm)/Al_0.7_Ga_0.3_P/GaP MOCVD		SiN	EBL	ICP-RIE (HBr/O_2_/He)			2018	[103]
PhC	GaP (300 nm)/Al_0.36_Ga_0.64_P/GaP (100) MOCVD	Directly bonding by 5 nm Al_2_O_3_ on both sides	HSQ	EBL	ICP-RIE (BCl_3_/Cl_2_/CH_4_/H_2_)		300 °C in vacuum to bond	2019	[59]
PhC	GaP (300 nm)/Al_0.1_Ga_0.9_P/GaP (100) MOCVD	Directly bonding by 5 nm Al_2_O_3_ on both sides	EBEAM RESIST	EBL	ICP-RIE			2022	[58]
PhC	GaP (300 nm)/GaAs MOVPE	BCB	HSQ	EBL	ICP-RIE			2023	[62]
PhC	GaP (300 nm)/Al_0.2_Ga_0.8_P/GaP (100) MOCVD	Directly bonding by 5 nm Al_2_O_3_ on both sides	HSQ	EBL	ICP-RIE (BCl_3_/Cl_2_/CH_4_/H_2_)		250 °C after bonding	2023	[24]
PhC	GaP (300 nm)/Al_0.8_Ga_0.2_P/GaP MBE	Membrane transfer	HSQ	EBL	ICP-RIE			2023	[104]
Quantum dots	Ligand exchange method, hot-injection method							2023	[105]
Resonator	GaP (200 nm)/Al_0.85_Ga_0.15_P (800 nm)/GaP MBE	Membrane transfer	HSQ	EBL	ICP-RIE (Ar/Cl_2_)			2014	[106]
Waveguide ring resonator	GaP (300 nm)/Al_0.36_Ga_0.64_P/GaP (100) MOCVD	Directly bonding by 5 nm Al_2_O_3_ on both sides	HSQ	EBL	ICP-RIE (BCl_3_/Cl_2_/CH_4_/H_2_ 10/10/5/14.5 sccm)		300 °C for 2 h to bond	2018	[60]
Waveguide	GaP (111) (400 nm)/AlGaInP/GaAs MOCVD	Directly bonding by 2 µm SiO_2_ on both sides	HSQ (540 nm)	EBL	ICP-RIE (N_2/_Cl_2_)			2021	[52]
Waveguide	GaP (160 nm)/Al_0.2_Ga_0.8_P (100 nm)/GaP (100) MOCVD	Directly bonding by 5 nm Al_2_O_3_ on both sides	HSQ	EBL	ICP-RIE chlorine-based			2021	[23]
Waveguide	GaP (001)/GaAs MOVPE	Directly bonding by two GaP membranes	SiN	EBL	ICP-RIE			2022	[51]
Waveguide	GaP (230 nm)/Al_0.7_Ga_0.3_P (1 µm)/GaP MOCVD	Suspended	EBEAM RESIST	EBL	ICP-RIE			2023	[107]
Waveguide	GaP (100) (600 nm)/AlGaInP/GaP MOCVD	NOA83H	RZJ304.10	UV lithography	ICP-RIE			2023	[13]
Waveguide ring resonator	GaP (100) (427 nm)/Al_0.8_Ga_0.2_P/GaP MBE	Membrane transfer	HSQ (∼100 nm)	EBL	ICP-RIE (N_2_ 3 sccm/Ar 6 sccm/Cl_2_ 1 sccm)			2018	[108]
Waveguide ring resonator	GaP (300 nm)/Al_0.38_Ga_0.62_P/GaP MOCVD	Directly bonding by 5 nm Al_2_O_3_ on both sides	HSQ	EBL	ICP-RIE (BCl_3_/Cl_2_/CH_4_/H_2_)			2020	[16]
Waveguide ring resonator	GaP (100) (427 nm)/Al_0.8_Ga_0.2_P/GaP MBE	Membrane transfer	HSQ	EBL	ICP-RIE			2021	[109]
Waveguide ring resonator	GaP (100) (250 nm)/Al_0.8_Ga_0.2_P/GaP MBE	Membrane transfer	HSQ	EBL	ICP-RIE			2022	[109]
Waveguide ring resonator	GaP (300 nm)/AlGaP (1 µm)/GaP MOVPE	Micro-transfer printing	SiO_2_	EBL	ICP-RIE			2022	[7]
Waveguide ring resonator	GaP (100) (427 nm)/Al_0.8_Ga_0.2_P/GaP MBE	Membrane transfer	HSQ (∼100 nm)	EBL	ICP-RIE (N_2_ 3 sccm/Ar 6 sccm/Cl_2_ 1 sccm)			2023	[110]
Waveguide ring resonator	GaP (40 nm)/BGaP (188 nm)/GaP (40 nm)/Si epitaxially grown	Membrane transfer	HSQ	EBL	ICP-RIE			2024	[111]
Waveguide/grating	GaP (300 nm)/AlGaP/GaP MBE	Membrane transfer	HSQ	EBL	ICP-RIE			2020	[112]

#### 3.1.6 Selection of hard masks

**Figure 7:**
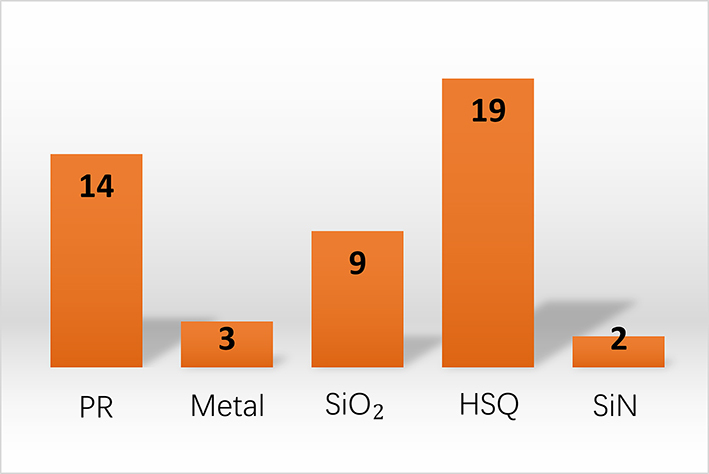
Choice of hard masks for the ICP-RIE process of GaP.

The choice of a hard mask for GaP etching depends on several factors, including compatibility with the etching process, selectivity, and the easiness to be removed after etching without damaging the underlying material. Here are some commonly used hard masks for GaP etching and their characteristics:

1. Silicon Dioxide
  SiO_2_ has excellent chemical resistance and thus high etching selectivity in chlorine-based etching recipes. It can be uniformly deposited using techniques like chemical vapor deposition. The removal of SiO_2_ is also straightforward using wet chemical etchants like hydrofluoric acid (HF). Here, we would extend the discussion to hydrogen silsesquioxane (HSQ), a commonly used EBL negative resist. HSQ is a silicon-based organic compound with a general formula of (HSiO_1.5_)_*n*_, known for its cage-like structures. Its chemical properties are comparable to SiO_2_ for GaP nanopatterning. To this end, 59.6 % in Figure 7 uses SiO_2_-based masks for ICP-RIE.
2. Silicon Nitride
  Similar to SiO_2_, Si_3_N_4_ provides high etch resistance and good selectivity, especially in plasma-based etching processes. Removal can be achieved using phosphoric acid at high temperatures with care.
3. Metal Masks
  Metals masks are especially useful for high-energy etch processes where dielectric or polymer materials might not withstand the conditions. Metal masks can be removed through wet etching processes specific to each metal, such as Al-etchant, Cr-etchant, and gold etchant. However, we found that Al and Cr could influence the etched sidewalls, which may negatively impact the nonlinear responses in GaP nanophotonic devices.
4. Photoresist
  A thick photoresist layer is sometimes useful in combination with other hard mask materials for added protection and pattern resolution. The mask must have a high selectivity to the etching process used for GaP, meaning it should provide enough protection before finishing etching the GaP. Good adhesion to the GaP substrate is also crucial to prevent mask peeling or undercutting during the etching process as chlorine-based gases can be rapidly diffused into the interfaces.

### 3.2 Devices

#### 3.2.1 Film stacks

GaP film stacks are integral components in various fields, notably optoelectronics and microelectronics, where their applications depend on the combination of multiple thin film layers, including at least one layer of GaP. This strategic layering is customized to provide the specific electrical, optical, or physical characteristics necessary for the application. GaP’s high refractive index makes it competitive for constructing optical devices such as waveguides, lasers, and photodetectors, particularly when stacked with materials like gallium nitride (GaN) or gallium arsenide (GaAs) to create specific optical properties. Furthermore, its use in creating quantum wells or dots in film stacks is crucial for manufacturing highly efficient optoelectronic devices and lasers. The design of these film stacks also considers thermal conductivity and resistivity to maintain stability during high-performance operations. Common fabrication techniques for GaP film stacks include MBE, CVD, and ALD, with the choice of method and specific design varying greatly based on the application. The precise control over thickness, composition, and layer order is important for achieving the desired performance of the device.

#### 3.2.2 Suspended GaP membrane devices

Suspended GaP structures, known for their excellent optical properties, high refractive index, and wide direct bandgap, are gaining more attention in nanotechnology and photonics. These structures, where a GaP layer is suspended in air or vacuum, minimize substrate interference, enhancing light–matter interaction and boosting device performance in applications like waveguides, resonators, and photonic crystals. Fabrication involves techniques such as EBL, ICP-RIE, and selective wet etching, often including undercutting a sacrificial layer. These structures’ durability during fabrication and use is crucial. Suspended GaP is utilized in sensors, offering high optical transparency, mechanical stability, and sensitive detection due to improved interaction with light. They also provide better thermal isolation for applications requiring heat control, such as in photonic integrated circuits. In quantum technology, suspended GaP structures are important for controlling quantum states and light interactions. However, fabricating these structures with precision without compromising material integrity and integrating them into larger systems remains significant challenges.

#### 3.2.3 Photonic crystals (PhCs)

Just like electronic band gaps in semiconductors, PhCs have photonic band gaps where certain wavelengths of light cannot propagate through the crystal, which property is crucial for creating devices like filters lasers, and waveguides. A high refractive index contrast between the GaP and the surrounding medium (like air or another material) enhances the photonic band gap effects, thus boosting efficient light control at small scales. One of the challenges is to create periodic structures without any defects at wish. This can be mitigated to photonic topological insulators (TI) – structures that exploit the principles of topological phases in photonics [65]. These TI systems can exhibit edge states that are robust against disorder and defects, akin to the edge states in electronic topological insulators. The robustness is due to the topological nature of the light modes, which are protected against scattering by imperfections or irregularities in the material [66].

#### 3.2.4 Metasurfaces

Metasurfaces, consisting of two-dimensional arrays of subwavelength structures on nanoscale substrates, are known for their ability to manipulate light’s amplitude, phase, and polarization efficiently and flexibly. The design of GaP metasurfaces focuses on engineering the size, shape, and arrangement of these structures to achieve specific optical effects, such as extraordinary transmission, polarization conversion, wavefront shaping, and anomalous reflection/refraction. These effects arise from the complex interaction between light and the nanostructures, enabling customized optical behaviors. GaP metasurfaces are important in applications like integrated photonics, optical sensing, imaging, and communication, enhancing the functionality of devices like lenses, beam deflectors, polarizers, and modulators. Their tunability through geometric or material modifications allows for application-specific optical responses and integration with other optical or semiconductor components, leading to compact, multifunctional photonic systems. However, challenges like complex fabrication, scalability, and broad spectral range optimization persist, requiring advancements in fabrication techniques, materials engineering, and computational design to maintain GaP metasurfaces’ leading role in nanophotonic innovation.

#### 3.2.5 Planar lightwave circuits (PLC)

GaP’s high refractive index and broad direct bandgap make it suitable for applications from the visible to near-infrared spectrum, ensuring strong light confinement and minimal loss. GaP waveguides are used in integrated photonic circuits, lasers, modulators, sensors, and nonlinear optical devices, excelling in effective light guiding and manipulation. Their fabrication typically involves advanced semiconductor processing like lithography, etching, and epitaxial growth. Precision in these processes is important for optimal optical performance. A key advantage of GaP waveguides is their significant nonlinear optical coefficients, useful for frequency conversion processes like second-harmonic generation. Integrating GaP waveguides with silicon photonics, which combines GaP’s light-emitting capabilities with silicon technology, is a thriving research area, offering more efficient, compact devices. Challenges include managing stress and defects, especially when integrating with materials like silicon. Current research focuses on refining fabrication, improving integration with other materials, and exploring new applications in areas like quantum computing and communications.

GaP ring resonators are essential in photonic circuits for their ability to manipulate light at a small scale. These resonators trap light at specific frequencies in a looped structure, enhancing light–matter interactions. Playing a key role in nonlinear optics for frequency mixing and ultrafast optical switching, GaP ring resonators find application across a range of areas, including in the creation of optical filters, modulators, sensors, and lasers. Their fabrication, involving precise techniques like lithography and etching, is key to determining resonant wavelengths and device efficiency. Commonly integrated with other photonic components, they play an important role in complex circuits. GaP’s nonlinear properties make these resonators effective in advanced processes like second-harmonic generation.

#### 3.2.6 Bound state in the continuum

A bound state in the continuum (BIC) refers to a peculiar quantum state where a bound state (which normally exists below the energy level of the continuum) is embedded within the continuum of energy states [67]. The concept of BIC originates from quantum mechanics and has implications in photonic and optoelectronic applications for PhCs, metasurfaces, and waveguide devices. In simpler terms, it is a localized state that doesn’t dissipate its energy, even though it exists in a range of energies where dissipation would be expected.

BICs can be observed in PhC structures or nanostructured materials where wave interference can create localized modes. Such states can lead to extremely sharp resonances in optical properties. For instance, in a GaP-based PhC, a BIC can result in highly localized and intense light fields.

## 4 Functional GaP devices

### 4.1 Linear GaP optical nanodevices

**Figure 8:**
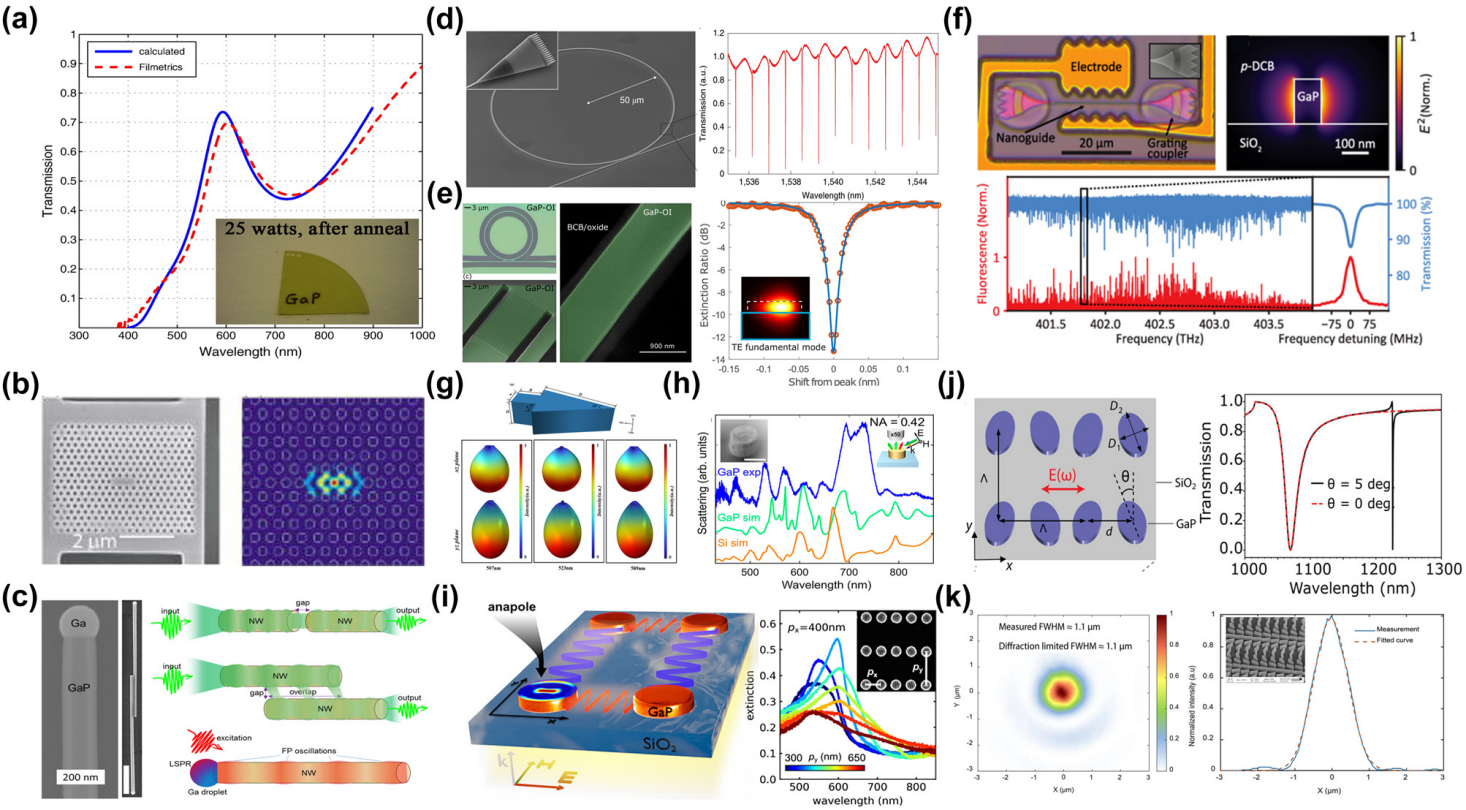
Linear optical responses on GaP devices. (a) Transmission test for GaP films fabricated with 25 W RF power after optimal annealing, inserting the films image. (b) SEM image of a fabricated GaP PhC membrane after undercut of sacrificial layer and simulation of electric field intensity inside the PhC cavity for the high *Q* mode. (c) SEM images of GaP nanowire (NW) with Ga droplet and GaP NWs coupled waveguides, scale bar 1 µm. The NWs coupling schematics include two aligned GaP NWs waveguiding system, waveguiding in parallel GaP NWs, and size-dependent resonant phenomena in individual GaP NWs. (d) SEM image of an uncladded ring resonator with integrated bus waveguide and grating couplers (inset). Measured transmission of a silica-cladded racetrack resonator (right figure). (e) Optical micrographs of GaP-OI optical waveguides with ring resonators fabricated using transfer-printed GaP layers and the finest resonance line for a wavelength of 1,534 nm. The red dots show the measurement data, and the blue curve is a fit using the model. The inset is a simulation of the field distribution for the fundamental TE mode in this ring resonator. (f) Optical image of a nanowaveguide interfaced with two electrodes. Simulated intensity profile of the TE mode in the nanoguide (right figure), measured extinction (blue) and fluorescence (red) spectra of hundreds of molecules the grating couplers on both sides of the nanoguide are covered by a polymer to shield them from irregularities in the p-DCB crystal. (g) Schematic illustration of the arrow-shaped GaP nanoantenna and 3D far-field radiation patterns at different wavelengths. (h) Experimental (blue) and simulated (green) scattering spectra of a GaP nanoantenna for *p*-polarized excitation. For comparison, the simulated scattering spectrum of the silicon nanoantenna with scaled geometrical parameters is shown in orange. The spectra are vertically displaced from each other for readability. The left inset in the SEM image of a large GaP nanoantenna and scale bar represents 500 nm. (i) Coupling mechanisms in arrays of anapole resonators, extinction spectra, and SEM images of 2D arrays of nanodisks. An infinite array of amorphous GaP nanodisks supported on a SiO_2_ substrate is illuminated by a broadband light source from the bottom in the *z*-direction. (j) GaP metasurface, comprising a square lattice (periods, Λ = 700 nm) of dimers formed by two elliptical cylinders with a relative tilting by an angle *θ* between their major axes. Simulated transmission spectra of *x*-polarized normally incident plane waves traveling through the metasurface with *θ* = 0° (red) and *θ* = 5° (black) in BIC. (k) Measured spot size of GaP metalens in the focal point at the wavelength of 450 nm and SEM image of the metalens. (a) Reproduced with permission [74]. Copyright 2011, Elsevier. (b) Reproduced with permission [5]. Copyright 2008, American Institute of Physics. (c) Reproduced with permission [113]. Copyright 2023, Molecular Diversity Preservation International. (d) Reproduced with permission [16]. Copyright 2020, Nature Research. (e) Reproduced with permission [7]. Copyright 2022, Optical Society of America. (f) Reproduced with permission [23]. Copyright 2021, American Phycical Society. (g) Reproduced with permission [114]. Copyright 2023, Optical Society of America. (h) Reproduced with permission [3]. Copyright 2022, American Chemical Society. (i) Reproduced with permission [90]. Copyright 2021, American Chemical Society. (j) Reproduced with permission [53]. Copyright 2020, American Chemical Society. (k) Reproduced with permission [6]. Copyright 2020, Nature Publishing Group.

GaP is particularly noteworthy for its high refractive index (*n* > 3) and transparency across a spectrum ranging from visible to infrared wavelengths (0.55–11 µm), rendering it an attractive material for light transmission applications. However, challenges arise due to the high absorption of visible light in films rich in Ga, which display a metallic luster, and those rich in *P*, which exhibit a deep brown color. Significant strides have been made to address these issues. The optical linear performance of GaP devices is summarized in Table 6. As shown in Figure 8(a), Gao et al. optimized GaP thin films prepared via RF magnetron sputtering through a high-temperature annealing process, achieving a high refractive index (*n* = 3.23) and low extinction coefficient (*K* = 0.029) at 633 nm [74]. Rivoire et al. utilized high-refractive-index GaP thin films to fabricate PhC nanocavities, observing resonances at wavelengths as low as 645 nm at room temperature with quality factors (Q) up to 1700 through cross-polarization reflectivity measurements. The schematic is illustrated in Figure 8(b) [5]. Bolshakov and his colleagues employed self-assembled nanowires as visible light waveguides, managing to keep coupling losses below 30 % for nanowires spaced by mere hundreds of nanometers through near-field coupling and the nanowire structure is presented in Figure 8(c) [113]. Makarov et al. integrated GaP nanowires directly into CsPbBr_3_ perovskite microcrystals, facilitating stable room-temperature lasing and broadband chemical tuning with emission wavelengths spanning 530–680 nm, and transmission distances exceeding 20 µm [86]. Billet et al. pioneered the fabrication of GaP photonic devices based on SiO_2_ through micro-nanofabrication techniques. This process involved bonding a GaP/Al_*x*_Ga_1−*x*_P/GaP heterostructure onto a SiO_2_-on-Si wafer, followed by the removal of the GaP substrate and the Al_*x*_Ga_1−*x*_P sacrificial layer. The resulting GaP grating couplers demonstrated high coupling efficiency of up to 4.8 dB, and ring resonators exhibited optical quality factors of 20,000 [60]. As shown in Figure 8(d), Seidler’s group entailed using direct wafer bonding to achieve integrated waveguides in the telecom C-band with losses of 1.2 dB/cm, comparable to those on silicon-on-insulator, and fabricating high-quality (*Q* > 10^5^) grating-coupled ring resonators [16]. Billet et al. employed micro-transfer printing to transfer GaP layers onto silicon dioxide wafers, leading to the creation of GaP-on-insulator ring resonators with *Q* factors up to 35,000 and extinction ratios exceeding 8.5 dB in the communication band, the structure and the fitting result can be seen in Figure 8(e) [7]. Shkarin et al. combined waveguides with single-molecule Stark shift monitoring to observe nanoscale charge fluctuations, achieving transmission losses as low as 0.5 dB/cm at a wavelength of 740 nm [23]. The pptical image of a nanowaveguide and simulated intensity profile of the TE mode in the nanoguide is presented in Figure 8(f). Process optimization has the potential to reduce transmission losses to 6 dB/cm [22]. Lastly, our implementation of an intermediate layer bonding technique for fabricating GaP-on-insulator (GaP-OI) photonic platforms enabled the realization of inverted ridge waveguide structures. Shallow etching of 1.8 µm wide waveguides at 1,550 nm wavelength resulted in propagation losses of 23.5 dB/cm [13] measured by the cutback method, marking a low-cost approach to enhancing manufacturability and scalability.

Subwavelength nanostructures exhibit an enhanced response to incident light, significantly augmenting their light absorption and scattering capabilities. Sugimoto et al. synthesized GaP nanospheres, demonstrating size-tunable Mie scattering from visible to near-infrared ranges, and providing modes of electromagnetic dipolar and multipolar resonances [91]. Makarov and his colleagues improved the forward and backward scattering efficiency of nanowire films formed by elongating nanoparticles in one direction, an advancement of substantial utility for second-order nonlinear visualization [96]. Liu et al. simulations of arrow-shaped GaP nanoantennas, which can be seen in Figure 8(g), revealed enhanced near-field electric field strength and unidirectional far-field scattering. The intensity of this scattering is highly sensitive to parameters such as antenna thickness and arrowhead angle and can be further amplified by increasing the number of nanoparticles in the array [114]. Maier’s group utilized MOCVD-deposited GaP single-crystal nanodisks. Under anapole excitation, these exhibited negligible absorption and confined various optical modes to the disk’s internal magnetic field at low scattering [54], which suggests a substantial enhancement of the antenna’s nonlinear effects across multiple wavelength regions. High-refractive-index amorphous GaP (a-GaP) nanopatches, deposited via RF sputtering on low-refractive-index glass substrates [84], displayed concentrated internal electric field energy with decreasing scattering, attributed to nonradiative or anapole-like excitation conditions. Grinblat et al. further observed that (100)-oriented GaP nanodisks on glass substrates presented high-field-confining radiative or quasi-nonradiative resonances. The radiative resonances had the highest scattering efficiency, and the nonradiative ones were the lowest. The radiation in the 800–1,200 nm wavelength range was contributed by magnetic dipoles (MD) and electric quadrupoles (EQ) [115]. Fedorov’s group obtained (111)-oriented GaP epitaxial layers on high-optical-contrast sapphire wafers. As shown in Figure 8(h), experimental (blue), simulated (green) scattering spectra of a GaP nanoantenna for *p*-polarized excitation and the simulated scattering spectrum of the silicon nanoantenna (orange) is compared, left inset in SEM image of a large GaP nanoantenna. Ellipsometry confirmed the GaP layer’s refractive index and absorption coefficients closely approximated those of bulk GaP crystals. Individual GaP nanoantennas supported both low-order and high-order optical resonances in the green spectrum of visible light [3]. Our group compared the scattering response of GaP slit nanoantennas under different excitation directions and found that lateral incidence anapole excitation conditions, involving multipolar resonances, resulted in stronger scattering and more concentrated central electric field energy [116]. By enhancing coupling through the slits of adjacent antennas, the GaP dimer structures achieved a far-field radiation efficiency of 99 % at resonant wavelengths, becoming an effective platform for surface-enhanced spectroscopy and nonlinear applications [4]. Krachmalnicoff et al. utilized dimers that exhibited responses of electric dipoles (ED) and magnetic dipoles (MD) in the visible range, enhancing the radiation decay rate of single emitters [88]. Hüttenhofer et al. conducted systematic studies on the coupling mechanisms in arrays of amorphous GaP nanodisk rectangles. The coupling mechanisms in arrays of anapole resonators, extinction spectra, and SEM images of 2D arrays of nanodisks are illustrated in Figure 8(i). These nanodisks supported anapole excitation at 600 nm, and the array’s maximum visible light extinction and GaP absorption were not achieved through the densest resonator packaging. Increasing the array period allowed collective effects in the spectrum to overlap with simulations of single-particle excitation, resulting in absorption enhancement up to 300 % compared to single disks [90]. The combination of anapole excitation and super-surface lattice resonance further enhanced the absorption of a-GaP in the visible spectrum [64]. Kuznetsov’s group utilized super-surfaces to support bound states in the continuum (BIC) under continuous medium modes. They constructed arrays of GaP cylinder dimers with elliptical cross sections as super-surface unit cells. When each unit of the cell was aligned parallel, wide resonances in the transmission spectrum at short wavelengths corresponding to BIC excitation were observed. The GaP metasurface and the simulated transmission spectra of *x*-polarized normally incident plane waves traveling through the metasurface are presented in Figure 8(j). At approximately 1,225 nm, narrow resonances corresponding to quasi-BIC excitation were seen, with an estimated *Q* factor exceeding 4,000 [53]. Melli et al. reported on the feasibility of manufacturing super-surfaces based on GaP thin films for optical applications in the visible range, with optimized grating super-surface parameters, achieving diffraction efficiencies of 40 %–50 % for the first-order diffraction peak numerically, the measured spot size of GaP metalens in the focal point at the wavelength of 450 nm and SEM image of the metalens is shown in Figure 8(k) [6]. Makarov’s group also observed this phenomenon in super-surfaces with rectangular cells. Slight asymmetry between adjacent cells opened radiative leakage channels, transforming symmetrically protected BIC into high-Q quasi-BIC. This subtle asymmetry provided effective coupling between incident waves and super-surface modes, allowing observation of sharp resonances in transmission (reflection) spectra, with experimentally observed *Q* factors around 100 [78].

### 4.2 GaP optical emitters and photoluminescent nanodevices

**Figure 9:**
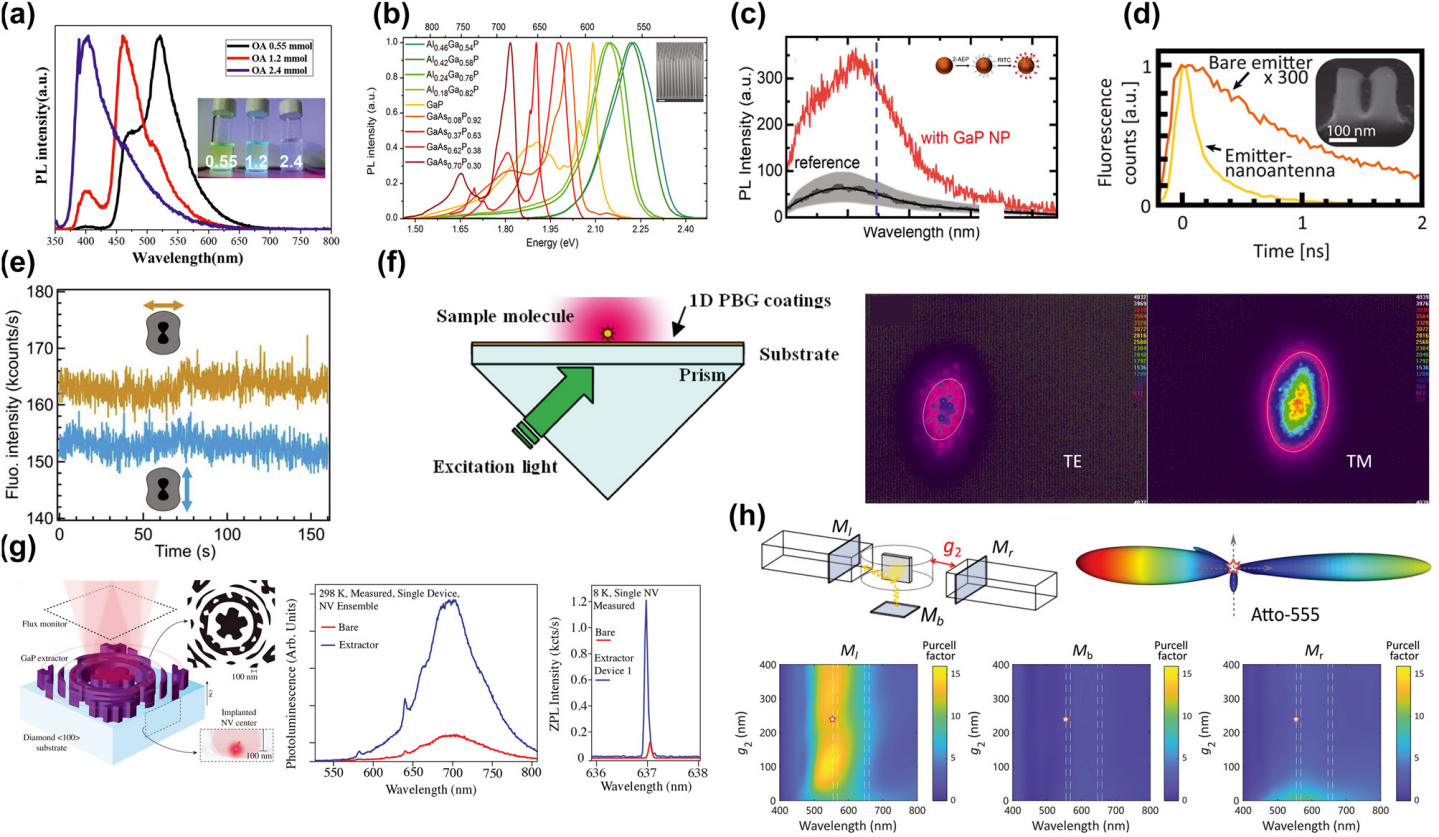
Photoluminescence using GaP devices. (a) Photoluminescence spectra of GaP QDs as a function of surfactant concentration. Inset is the PL image of 520, 460, and 400 nm emission from GaP QDs solutions obtained on a UV lamp. (b) Photoluminescence spectra of Al_*x*_Ga_1−*x*_ P and GaAs_*y*_P_1−*y*_ ternary compound wires, showing the tunability of the emission wavelength. Inset is an SEM picture of GaP/Al_0.4_Ga_0.6_P core/shell nanowires in a nanoimprint pattern (tilting angle 70°), and the scale bar corresponds to 1 μm. (c) PL spectra of a GaP NP with RITC (red solid curve) and a reference sample (gray solid curve). The black dashed curve is a spectrum after smoothing to eliminate fluctuations by interference in a BP flake. Gray shaded region in the reference spectrum indicates fluctuation of the PL intensity in 25 measured spots on a GaP flake. Inset is a schematic of the preparation procedure of a GaP NP with RITC. (d) Fluorescence emission decay curves with a 35 nm gap GaP dimer and bare emitter. Inset is the SEM image of GaP dimer, the scale bar of SEM is 100 nm. (e) Fluorescence intensity-time traces recorded on a 1.4 μM solution of Alexa Fluor 647 with 200 mM methylviologen on a topologically optimized GaP nanoantenna with the excitation polarization parallel (orange) or perpendicular (blue) to the 30 nm bowtie nanogap. The binning time is 100 ms. (f) Fluorescence enhancer setup with multilayer 1D PBG coatings on the surface of the glass substrate. Fluorescence images captured by the CCD camera for 1D PBG sample with TE and TM enhancement (right figure). (g) Schematic of the GaP photon extractor designed via topology optimization. The broadband PL emission by NV ensemble from an extractor device (blue) versus the bare diamond interface (red) and the single NV ZPL emission on sample B from an extractor device (blue) versus the bare diamond interface (red). (h) Schematics of GaP waveguide collection and directional radiation for fluorophores Atto-555 (labeled as star) in 3D presentation with *g*_2_ = 240 nm (red and blue, respectively, indicate strong and weak radiation). Purcell factors from left, bottom, as well as right side when changing the distance *g*_2_ away from the second waveguide. Three monitors *M*_*I*_, *M*_*b*_, and *M*_*r*_ are placed separately on the left, bottom, and right side of the nanodisk to estimate the emitted powers. (a) Reproduced with permission [105]. Copyright 2023, Elsevier. (b) Reproduced with permission [95]. Copyright 2013, American Chemical Society. (c) Reproduced with permission [91]. Copyright 2023, Wiley. (d) Reproduced with permission [4]. Copyright 2017, American Chemical Society. (e) Reproduced with permission [87]. Copyright 2024, American Chemical Society. (f) Reproduced with permission [75]. Copyright 2011, Optical Society of America. (g) Reproduced with permission [112]. Copyright 2020, Optical Society of America. (h) Reproduced with permission [116]. Copyright 2020, Wiley.

GaP, a member of the III–V family of semiconductor materials, exhibits spontaneous emission from ultraviolet to blue-green light in the 250–500 nm wavelength range, primarily attributable to its high absorption coefficient at short wavelengths. The photoluminescence enhancement using GaP devices is shown in Table 7. Thin films composed of rough, porous GaP nanocrystals demonstrate more pronounced fluorescence peaks at specific wavelengths of 397 nm, 410 nm, 453 nm, and 470 nm compared to the original GaP powder [73]. When gold (Au) and silver (Ag) nanoparticles are added, the photoluminescence (PL) intensity of GaP can be enhanced up to 5.6 times and 14.5 times, respectively [68]. GaP thin films with a surface roughness not exceeding 1 nm display band-edge PL characteristics at room temperature [2]. Colloidal GaP quantum dots, synthesized through a hot injection method and optimized precursor combination, achieve controllable emission in the 400 nm–520 nm range, particularly exhibiting high PL quantum yields of 35–40 % and a full width at half maximum (FWHM) of 75 nm in the green region, which is illustrated in Figure 9(a) [105]. Moreover, pure hexagonal crystal structure GaP nanowires, exhibiting direct band gap features, also demonstrate significant PL at 594 nm. By doping with aluminum or arsenic, emission wavelengths within the 555–690 nm range can be tuned. The photoluminescence spectra are presented in Figure 9(b) [95]. These findings highlight GaP’s tremendous potential in optoelectronic and luminescent applications.

**Table 6:** Optical linear performance of GaP devices.

Structure	Working	Transmission (T)/reflection (R)	Power	Year	Reference
	wavelength	Scattering cross section (SCS)			
Film	633 nm	Coefficient *K* = 0.029		2011	[74]
PhC cavity	645–750 nm	*Q* up to 1700		2008	[5]
Nanowires 250 nm diameter	530–680 nm	*T* ∼ 35 %, coupling losses <30 % (sims.)	1 W/m^2^	2022	[113]
Nanowires	700–1,300 nm	Efficient light scattering in forward and backward directions, useful for the SHG visualization	50 mW	2020	[96]
Nanoparticle	Visible range	Scattering peaks in different Mie modes (magnetic, electric dipole, and quadrupole)		2023	[91]
Arrow-shaped antenna	Visible range	Unidirectional scattering		2023	[114]
Nanodisk	Visible range	650 nm response		2022	[3]
	Visible range	Scattering tilts in anapole modes		2021	[117]
				2023	[116]
	400–1,200 nm	Scattering in anapole modes, electric quadrupole, magnetic dipole		2021	[115]
	600–1,000 nm	Scattering minima at anapole excitation	600 mW	2020	[54]
Nanopatch	600–1,500 nm	Scattering dip in anapole-like excitation	600 mW	2020	[84]
Dimer	Visible range	Scattering efficiency of 99 % at resonance	2.5 mW/cm^2^	2017	[4]
		Scattering in magnetic and electric dipole		2024	[88]
Waveguide (200 nm diameter, 100 × 160 nm^2^, 800 × 300 nm^2^ cross section)	530–680 nm	Outcoupling light long-range guiding over distances of more than 20 μm		2020	[86]
	740 nm	Loss coefficient of about 0.5 dB/cm		2021	[23]
	1,534 nm	Extinction ratio is more than 8.5 dB, *Q*-factor of 40,100		2022	[7]
	1,550 nm	1.2 dB cm^−1^ loss	∼100 mW	2020	[16]
	1,550 nm	6 dB cm^−1^ loss	100 W	2023	[22]
	1,550 nm	23.5 dB cm^−1^ loss		2023	[115]
Grating couplers	1,553 nm	Peak coupling efficiency is as high as 4.8 dB	3.7 mW	2018	[60]
Grating metasurface	520 nm	Diffraction efficiency for the first diffracted order 40 %–50 %		2020	[6]
Metasurface	Visible range	Absorption enhancement up to 300 %		2021	[90]
	550 nm, 640 nm	Transmission minima	100 mW/cm^2^	2021	[64]
	1,225 nm	Transmission minima for BIC	37 mW/360 mW	2020	[53]
	800–860 nm	Transmission minima for BIC		2021	[78]

Unlike metallic plasmonic structures, GaP exhibits exceptional fluorescence enhancement in visible and near-infrared spectra with its high refractive index and low absorption losses. GaP nanoantennas, with their electric and magnetic multipolar resonances, offer extensive degrees of freedom for customizing light–matter interactions. For instance, GaP nanospheres fabricated by mechanical milling and pulsed laser melting exhibit resonance in both dipole and higher-order modes within the visible spectrum, significantly enhancing the surface molecule’s fluorescence Purcell factor, which is illustrated in Figure 9(c) [91]. On the other hand, GaP antenna structures made using semiconductor processes, despite having low *Q*-factors and small modal volumes, broaden the working spectrum range and reduce the volume of the field enhancement area. Notably, a GaP dimer with a 35 nm gap can enhance the fluorescence emission of dyes located in the nanoantenna gap by up to 3,600 times and significantly shorten the fluorescence lifetime by at least 22 times as shown in Figure 9(d) [4], while achieving a 30-fold increase in the Purcell factor in the gap region [88]. The integration of GaP nanoantennas with two-dimensional semiconductor transition metal WSe_2_ achieves up to 10^4^-fold enhancement in photoluminescence [83], with an average fivefold increase in quantum efficiency [85], revealing the role of dark exciton reservoirs and Auger processes, offering a platform for efficient quantum light generation. Additionally, slotted antennas attract attention for their high electric field enhancement in nonradiative resonance modes [117]. Slit antennas based on a topologically optimized design of GaP can increase fluorescence brightness by 90 times, with optical confinement within an extremely small 200 zeptoliter (10^−21^ L) detection volume, 5,000 times lower than the confocal diffraction limit, which is presented in Figure 9(e) [87]. GaP elliptical cylinder arrays supporting quasi-bound states in the continuum (QBICs) resonances in hyper-surfaces offer nearly three orders of magnitude electric field intensity and extremely high radiative enhancement in continuous media [115]. Suspended one-dimensional GaP hyper-surfaces exhibit a nearly flat photonic band over a 10° half-angle at ∼590 nm when integrated with cadmium selenide nanosheets [79], enabling the coupling of emerging quantum emitters into hyper-surfaces. One-dimensional photonic bandgap structures composed of GaP thin films enhance fluorescence collection efficiency in total internal reflection imaging systems as shown in Figure 9(f) [75]. Additionally, GaP PhC cavities, due to their high-Q and small modal volume, show potential in the quantum light source domain. In experiments where nano-diamonds with single nitrogen-vacancy centers are placed on the cavity surface, a 12.1-fold increase in the Purcell factor at the zero-phonon line (ZPL) emission is observed [102]. Integration with silicon-vacancy (SiV) centers in diamonds using stamp transfer techniques results in a threefold decrease in resonant lifetime [104]. A GaP-diamond dielectric structure designed inversely, coupled to a single near-surface nitrogen vacancy center, enhances the photon extraction bandwidth by 14 times as shown in Figure 9(g) [112].

### 4.3 Second-order nonlinear optical nanodevices

#### 4.3.1 Second-harmonic generation

SHG is a coherent nonlinear process that transforms two photons of frequency *ω* into a single photon of frequency 2ω. On a macroscopic scale, SHG, a second-order process, necessitates noncentrosymmetric crystal symmetry in the excited structure to produce a nonzero SHG signal. However, inhomogeneities in the electric field or the material itself at the nanoscale can elicit high second-order nonlinear responses, even in structures with centrosymmetric lattices. Utilizing this property, various structured plasmonic nanoantennas have achieved second-harmonic conversion efficiencies of up to 10^−6^ % over the past decade. Nevertheless, the substantial absorption in metallic nanostructures at plasmonic resonance severely restricts the amount of power transferable to the nanosystem without causing material damage or altering its refractive index, thereby limiting the achievement of higher *η*_SH_. To tackle this, recent developments in low-loss dielectric nanoantennas composed of silicon, germanium, and AlGaAs have demonstrated encouraging results in both second- and third-harmonic generation. Specifically, AlGaAs nanoantennas have attained *η*_SH_ values of about 10^−3^ % at near-infrared SH wavelengths. However, this approach has not yet been widely applied to generate efficient SH light within the visible spectrum, primarily due to the relatively higher absorption of most high refractive index dielectrics at these wavelengths. In contrast, the absorption coefficient of GaP at optical frequencies is significantly lower – more than three orders of magnitude – compared to materials like gold (Au), silver (Ag), and AlGaAs. This makes GaP nanoantennas a promising candidate for visible SHG, offering a potential pathway to overcome this spectral limitation.

**Figure 10:**
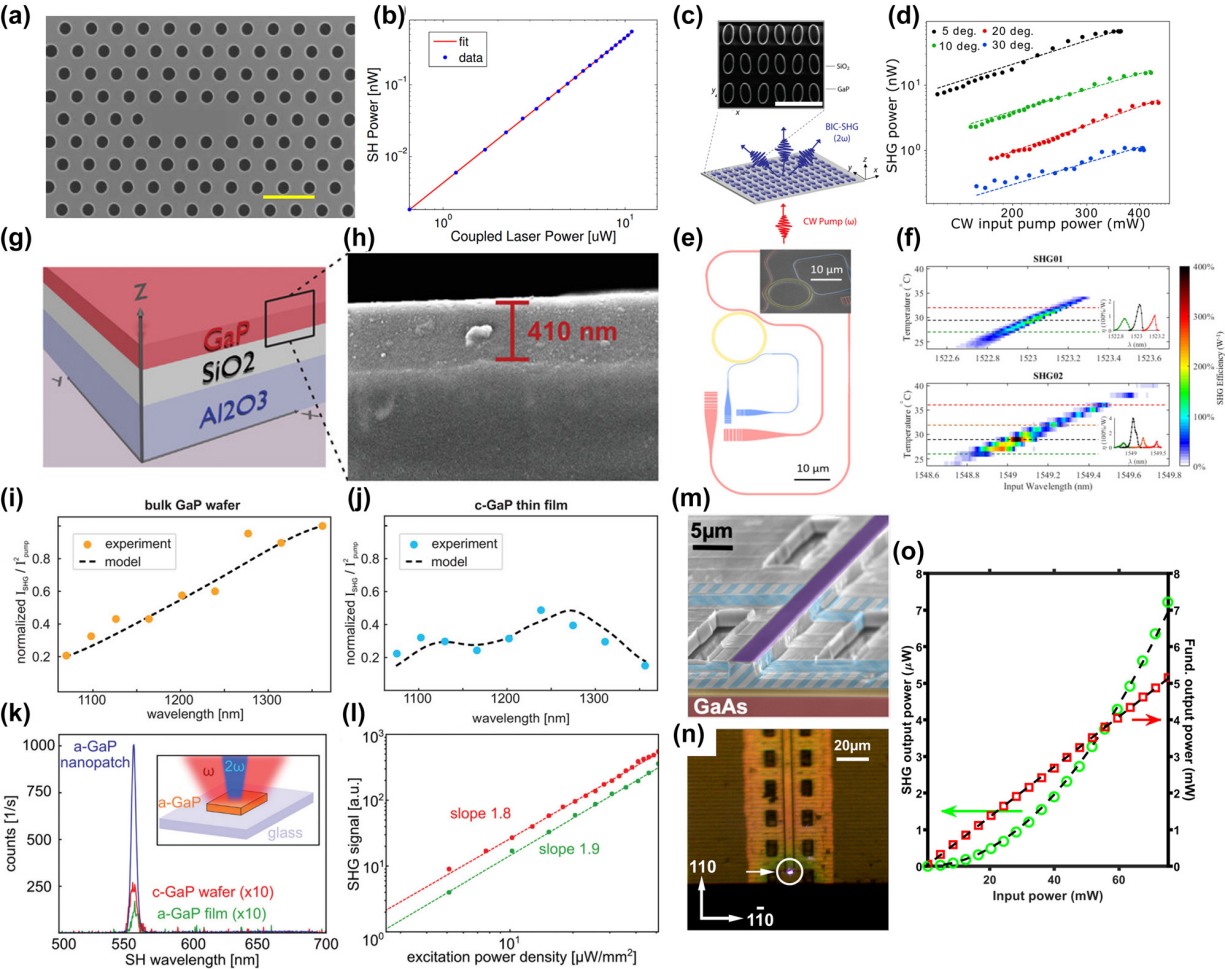
Up-converted second-order nonlinear all-optical effect using GaP devices. (a) SEM image of the PhC structure (b) second-harmonic power as a function of fundamental wavelength power coupled into the cavity. (c) Schematic and SEM image of the metasurface sample. (d) SHG power as a function of input CW pump power for various angles of the dimer. (e) The on-chip layout of the nonlinear ring resonator. Inset: SEM image of a fabricated GaP SHG device. (f) SHG conversion efficiency of two different devices as a function of both temperature and input wavelength devices. Scheme (g) and SEM image (h) of the stack films sample. Power normalized efficiency of the SHG intensity for the bulk [111] sample (i) and the c-GaP film (j). (k) Spectral SHG response for the c-GaP wafer (red), a-GaP film (green), and a single a-GaP nanopatch (blue). Inset: Sketch of the SHG measurement of a GaP nanopatch on a glass substrate. (l) Power dependence for the c-GaP wafer and a-GaP film at double logarithmic scale. (m) A bird’s-eye view of a suspended OP-GaP waveguide acquired in an SEM. (n) Visible-light top view of the waveguide (right), acquired *in situ* during optical measurements. (o) Plot of the transmitted power for the fundamental and the second harmonic as a function of input pump power. (a)–(b) Reproduced with permission [101]. Copyright 2009, Optical Society of America. (c)–(d) Reproduced with permission [53]. Copyright 2020, American Chemical Society. (e)–(f) Reproduced with permission [108]. Copyright 2018, Optical Society of America. (g)–(j) Reproduced with permission [8]. Copyright 2023, Wiley. (k)–(l) Reproduced with permission [84]. Copyright 2020, Royal Society of Chemistry. (m)–(o) Reproduced with permission [51]. Copyright 2022, American Chemical Society.

Rivoire and colleagues pioneered research into the generation and enhancement of second-harmonic generation (SHG) in GaP [101], [118]. The SEM image of the PhC structure is shown in Figure 10(a), while the second-harmonic power as a function of fundamental wavelength power coupled to the cavity is presented in Figure 10(b). Utilizing a continuous-wave laser at a 1,550 nm wavelength and a power of 6 μW, they successfully observed SHG in micro- and nano-fabricated PhC cavities, achieving an impressive efficiency of 430 % W^−1^. This breakthrough was attributed to the effective spatial overlap of fundamental and second-harmonic fields within the cavity, satisfying phase-matching conditions. They further enhanced this system’s versatility by tuning the cavity resonance frequency, creating a source adjustable within a 10 nm range. This initial work steered further focus on GaP, inspiring the design of diverse micro- and nanostructures to amplify SHG signals. Building on this momentum, Sanatinia et al. used nanosphere lithography to create periodic arrays of GaP nanocolumns on undoped GaP substrates, finding a strong correlation between SHG intensity and column diameter [92], [93]. Achieving a conversion efficiency of 2 × 10^−7^ % under a pulsed laser with a central wavelength of 840 nm and power of 2.8 mW, they provided valuable insights for future designs. In 2016, David P. Lake and colleagues developed a novel microdisk structure, coupling light of 1,550 nm wavelength into the disk using a tapered fiber at a specific pump power of 0.36 mW [28]. By employing intracavity photothermal temperature tuning, they fulfilled dual resonance conditions between high quality factor modes at both infrared and visible wavelengths in the GaP microdisk, attaining a conversion efficiency of 3.8 × 10^−4^ mW^−1^. Following these developments, Cambiasso et al. introduced GaP all-dielectric nanoantennas as a formidable nanophotonic platform for surface-enhanced SHG, showcasing minimal losses in the visible range [4]. Under a pulsed laser with a 910 nm central wavelength and 200 GW/cm^2^ power density, they attained a conversion efficiency of 0.0002 %, a figure three orders of magnitude higher than that of bulk materials and unprecedented for single nano-objects in the optical domain. Unlike microresonators that rely on high quality factors for field enhancement, these nanoantennas utilized small mode volumes with lower quality factors, expanding the operational spectral range and minimizing the field-enhanced area. The potential of high refractive index dielectric nanoantennas as an alternative to plasmonic counterparts for light concentration and manipulation at the nanoscale began to gain recognition. GaP nanoantennas, for instance, exhibit exceptionally low losses and high nonlinearity in the visible range, support both electric and magnetic resonances, and are compatible with CMOS fabrication technology. Remesh and colleagues created single GaP nanoantennas facilitating efficient SHG under Mie resonance conditions [89]. These nanoantennas support light-induced Mie resonances in the visible and near-infrared range, offering broad wavelength tunability and the ability to support both strong electric and magnetic modes, leading to distinctive optical properties. Remarkably, under a pulsed laser with a central wavelength of 790 nm and a power density of 1 GW/cm^2^, the conversion efficiency for the second harmonic reached 56 %, underscoring the potential of dielectric nanocavities in creating coherent nonlinear nanodevices. Complementing these findings, McLaughlin and associates demonstrated the simultaneous generation of second- and third-harmonic signals from GaP microdisks, boasting high quality factors of up to 60,000 under telecommunications wavelength pumping [82]. By analyzing the harmonic outputs’ power scaling and calculating nonlinear cavity mode coupling factors, they explored the roles of direct and cascaded sum-frequency generation processes in the third-harmonic signal. Observing a second-harmonic conversion efficiency of 5.9 × 10^−2^ % mW^−1^ at a continuous-wave laser wavelength of 1,557 nm and power of 8.7 mW, they confirmed the significance of both processes, despite GaP’s relatively higher material absorption at the third-harmonic wavelength, with their relative magnitudes being closely dependent on the detuning between the cavity modes and the second-harmonic wavelength.

Despite advancements in optical mode and material engineering, the realization of all second-harmonic generation (SHG) devices based on nanoantennas has been confined to high-intensity, pulsed states, often involving femtosecond lasers. This limitation restricts their practical application scope and affects efficiency due to bandwidth mismatches with high quality factor modes. To surpass these constraints, various structures have been developed to bolster resonance or refine phase matching for improved SHG conversion efficiencies. Notably, resonant metasurfaces present an effective platform for augmenting nonlinear optical processes, capable of generating substantial local electromagnetic fields while easing phase-matching requirements. Anthur et al. have made significant strides by engineering a high quality factor (*Q* ∼ 2000) photonic mode in a bound state in the continuum to enhance SHG. They attained SHG conversion efficiencies of 5 × 10^−5^ W^−1^ and 0.1 % W^−1^ under continuous and pulsed laser excitation, respectively, bringing metasurface-based SHG closer to practicality [53]. Figure 10(c) shows the schematic and SEM image of the metasurface sample, and the SHG power as a function of input continuous wave (CW) pump power for various angles of the dimer is shown in Figure 10(d). Additionally, waveguides, crucial in integrated photonic circuits for their strong light confinement and extensive phase-matching adaptability, have been designed to amplify SHG nonlinear processes. Anthur and his colleagues crafted GaP nanowaveguides, achieving phase-matched SHG by varying waveguide widths and pump wavelengths [52]. The precise measurement of the crystalline axial orientation in GaP thin films, vital for SHG modeling, facilitated an SHG conversion efficiency of 0.4 % W^−1^ cm^−2^ under continuous wave pumping. Another integral component in these circuits is the microring resonator, known for its high quality factors, broad wavelength selectivity, and tunability, as well as low loss. Logan and his team successfully achieved SHG conversion from 1,550 nm to 775 nm in GaP on an oxide-integrated photonic platform, reaching an SHG efficiency of 400 % W^−1^ [108]. This was achieved on a platform comprising a dual-resonant, phase-matched ring resonator. The schematic and SEM image of the on-chip layout can be seen in Figure 10(e), and the SHG conversion efficiency of two different samples is illustrated in Figure 10(f). Furthermore, they introduced a technique using hydrogen silsesquioxane (HSQ) electron beam exposure for precise postresonance wavelength tuning of GaP microring resonators [109]. This method allowed for fine adjustments to multiple devices on a single chip, targeting specific wavelengths with around 30 pm precisions. These devices demonstrated SHG in communication to near-infrared bands, showcasing a quality factor of about 10^4^.

In integrated photonic platforms, waveguides made from high refractive index materials like GaP are often paired with claddings of low refractive index materials. Typically, high-quality GaP crystal films are grown on GaAs substrates, which possess a higher refractive index, using advanced techniques such as MBE or MOCVD. Conventional manufacturing involves transferring GaP films onto substrates with lower refractive index materials like silicon dioxide. However, some researchers have developed GaP films or suspended structures on low refractive index substrates through alternative deposition or etching processes. Tilmann et al. undertook a comparative study of the harmonic generation performance between amorphous (a-GaP) and crystalline GaP (c-GaP) nanofilm [8]. The schematic and SEM image of the stack films sample is illustrated in Figure 10(g) and (h). Interestingly, the amorphous films, deposited on glass substrates using RF sputtering, demonstrated that epitaxially grown GaP films were on par with bulk crystals in terms of second-harmonic generation (SHG) efficiency across a range of pump wavelengths from 1,060 nm to 1,370 nm. Their findings, in congruence with nonlinear simulations, revealed comparable dispersion and magnitude of second-order nonlinear optical polarizability to bulk materials, which can be seen in Figure 10(i) and (j). They used nonlinear scattering theory and the transfer matrix method (TMM) to ascertain the second-order nonlinear magnetization rate within this wavelength spectrum. Notably, they showed that a-GaP films, especially for odd-order nonlinear processes, surpassed crystal samples in efficiency and offered simpler processing and manufacturing flexibility. In another study, they explored depositing amorphous GaP on silicon dioxide substrates and its nanostructuring [84]. This research indicated that a-GaP possesses optical properties akin to c-GaP, particularly noted for its low-loss transparency above 650 nm wavelengths. The SHG response of individual a-GaP nanopatches was found to be enhanced by more than two orders of magnitude compared to unstructured films, which can be seen in Figure 10(k), highlighting a-GaP grown by RF sputtering deposition as a promising material for nonlinear nanophotonics applications, presenting cost-effective and versatile substrate options compared to traditional c-GaP wafer-based techniques. Figure 10(l) shows the power dependence for the c-GaP wafer and a-GaP film at a double logarithmic scale. Khmelevskaia et al. developed a method to directly grow high-quality crystalline GaP on sapphire substrates for nonlinear all-dielectric nanophotonics using MBE [78]. They successfully nanostructured the GaP layer on sapphire (0001) substrates using metal masks and chemical plasma etching, creating GaP metasurfaces with outstanding linear and nonlinear optical properties. These metasurfaces demonstrated support for near-infrared bound states in the continuum (BIC) with an experimental quality factor (*Q* factor) of about 100, showing over tenfold SHG enhancement around the quasi-BIC state compared to nonresonant incident wavelengths. Pantzas et al. introduced a novel process for creating orientation-patterned GaP (OP-GaP), demonstrating the design and fabrication of suspended, air-clad OP-GaP shallow ridge waveguides for SHG [51]. Figure 10(m) is a bird’s-eye view of a suspended OP-GaP waveguide acquired in an SEM, and Figure 10(n) shows the visible-light top view of the waveguide acquired *in situ* during optical measurements. They recorded a remarkable SHG conversion efficiency of 200 % W^−1^ under continuous-wave pumping at 1,596 nm with a power of 70 mW, as shown in Figure 10(o). Furthermore, Fedorov and colleagues utilized MBE to cultivate GaP nanowires on silicon (111) substrates, later encapsulating them in PDMS [96]. The unique optical response of this structure, derived from GaP’s high second-order nonlinear susceptibility and the optical resonance effects of the nanowires, was enhanced by a specially designed tapered structure, elevating SHG efficiency. In their experiments with a pulsed laser at a central wavelength of 1,048 nm, they achieved an SHG conversion efficiency of 10^−4^. Furthermore, the SHG performance of InGaP-OI has been demonstrated on a wire waveguide aligned with a crystallographic axis, reaching an experimental conversion efficiency of 12 %/W where the limitation is pointing toward the propagation loss [119]. The second-harmonic generation with an efficiency of 71,200 ± 10,300 %/W on InGaP-OI [120] paved the way for other up-converted as well as down-converted processes with the advanced *d*_14_ of 220 pm/V. The SHG performance of GaP devices are summarized in Table 8.

**Table 7:** Photoluminescence enhancement using GaP devices^b^.

Structure	Excitation wavelength	Emission wavelength	Performances	Power	Year	Reference
200 nm thick film	360 nm	548.605 nm (2.26 eV) (A.F.)			2017	[13]
8 μm thick film	390 nm	511 nm (A.F.)			2015	[7]
Nanostructured thin film	300 nm	397 nm, 410 nm, 453 nm, 470 nm (A.F.)	PL enhancement 2× than powder particles		2010	[77]
Nanowires	405 nm	555–690 nm emitter (A.F.)			2013	[44]
Quantum dots	500 nm	400–520 nm emitter (A.F.)			2023	[58]
1D PhC band gap	532 nm	Qdots (R) 625 ITK carboxyl	PL enhancement 69× than bare glass		2011	[78]
**PhC cavities**	**532 nm**	**Single NV center** ^**a**^	**Purcell-factor 12.1×**	25 nW/750 nW	**2010**	[79]
		**Silicon-vacancy centers** ^**a**^	**3 × lifetime reduction for *Q* = 4,100 cavity**	100 mW/cm^2^	**2023**	[57]
Nanospheres	532 nm	Rhodamine B isothiocyanate	PL enhancement 69 × than a flake at the MQ wavelength		2023	[91]
Slotted nanodisk	730 nm	Electric dipole source	Electric field enhancement 1,063 (sims.)		2021	[38]
	635 nm	Alexa Fluor 647	PL enhancement 93×	∼130 nW	2024	[34]
**Inverse-designed photon extractors**	**532 nm**	**Nitrogen-vacancy centers** ^**a**^	**PL 14 × broadband enhancement**		**2020**	[112]
Dimers (35 nm gap 50 nm gap 20 nm gap)	633 nm	Star635p	PL enhancement 3,600×, lifetime reduction of at least 22×	12.4 mW	2017	[28]
	638 nm	2D WSe_2_	PL enhancement 10^4^ times	37 mW/360 mW	2019	[29]
	**725 nm**	**2D WSe**_**2**_^**a**^	**PL enhancement 10** ^**4**^ **times**	9 mW	2021	[31]
	625 nm	Abberior cage 635	Purcell-factor 30×	50 mW	2024	[76]
Waveguide	530 nm	Atto-555	PL enhancement 22.9 × (sims.)	0.6 W/cm^2^	2023	[74]
**Waveguide coupled nanodisk**	**640 nm**	**NV centers** ^**a**^	**Maximum collection efficiency of ∼22 %**		**2014**	[106]
**Grating metasurface**	**532 nm**	**CdSe nanoplatelets** ^**a**^	**Flat photonic band at ∼590 nm, *Q* > 500**		**2023**	[24]
Slotted metasurface	850–900 nm	Electric dipole source	Electric field enhancement 6 × 10^4^ radiative enhancement > 10^4^ (sims.)	100 mW/cm^2^	2021	[75]

^a^Single photon applications. ^b^A.F. stands for autofluorescence. The meaning of the bold values is single photon applications.

**Table 8:** Second-harmonic generation using GaP devices.

Pump wavelength (nm)	Structure	Crystalline 100 or 111	*d*_14_ (pm/V)	Quality factor	Input power	Efficiency	Pulse/CW	Year	Reference
1,200	Film	100/amorphous	74 (1313 nm)		30–200 mW	Comparable to a bulk wafer	Pulse	2023	[8]
1,200	Metasurface	100^a^	70 (1,000 nm)	2000	10^−2^/10^−6^ GW/cm^2^	4 × 10^−5^/2 × 10^−7^ (0.1 % W^−1^/5 × 10^−5^ % W^−1^)	Pulse/CW	2020	[53]
800–860	Metasurface	111	70		6 mW	100× than 200 nm polycrystalline thin film	Pulse	2021	[78]
1,550	Microdisk	100		FH 1.1 × 10^5^ SH 10^4^	0.35 mW	3.8 × 10^−4^ mW^−1^	CW	2016	[28]
1,557	Microdisk	100		6 × 10^4^	8.7 mW	5.9(1.6) × 10^−2^ % mW^−1^	CW	2022	[82]
910	Nanoantenna	100			200 GW/cm^2^	0.4 % MW^−1^/0.0002 %	Pulse	2017	[4]
790	Nanodisk	100			1 GW/cm^2^	56 %	Pulse	2019	[89]
1,120	Nanopatch	100/amorphous			0–102 μW mm^−2^	>100 × than a 260 nm amorphous thin film	Pulse	2020	[84]
840	Nanopillar	100	159 (852 nm)		2.8 mW	2 × 10^−9^	Pulse	2012	[92]
1,048	Nanowire	111	70 (1,000 nm)		N.A.	10^–4^	Pulse	2020	[96]
1,500	PhC	100			11 µW	430 % W^−1^	CW	2010	[101]
1,550	Ring resonator	100	100	FH 4.07 ± 1.07 × 10^4^ SH 1.68 ± 0.32 × 10^4^	3 mW	400 % W^−1^	CW	2018	[108]
1,528–1,552	Ring resonator	100		3 ± 1.7 × 10^4^	N.A.	Close to quasi-phase-matched condition	CW	2022	[109]
1,283.5	Waveguide	111	50 (1550 nm)		12.4 mW	0.4 % W^−1^cm^−2^	CW	2021	[52]
1,596	Waveguide	001	50 (1550 nm)		70 mW	200 % W^−1^	CW	2022	[51]

^a^15° with respect to the normal to the wafer surface.

#### 4.3.2 Sum frequency generation

**Figure 11:**
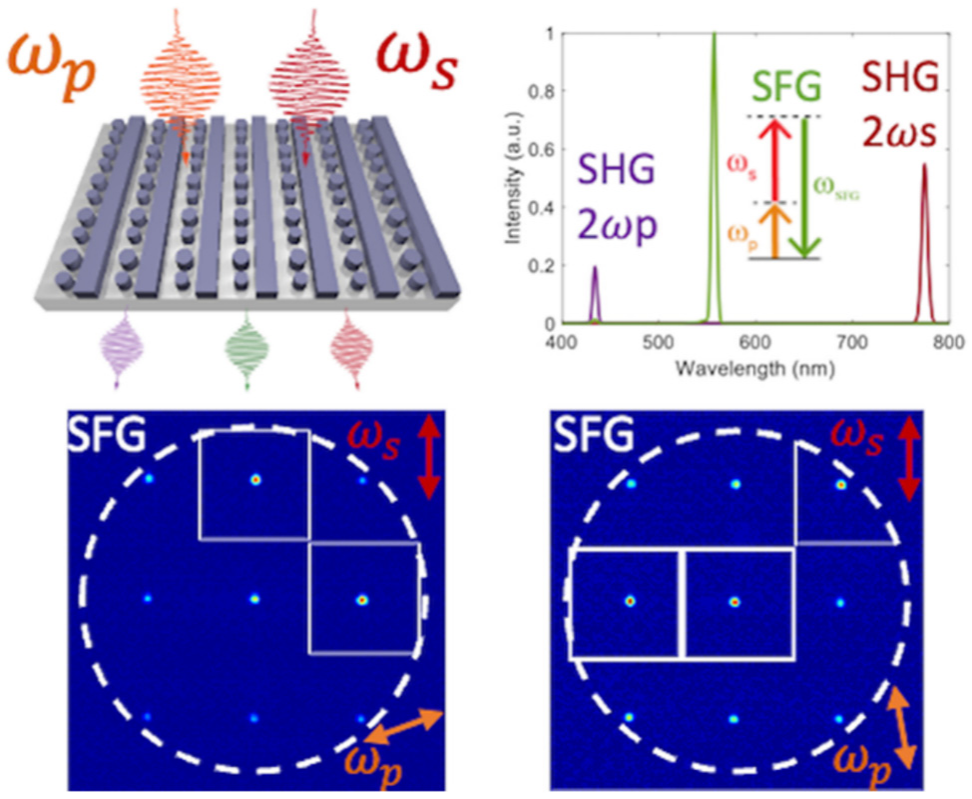
Sum frequency generation using GaP metasurfaces. Schematic of designed GaP metasurface, simultaneously illuminated by pump (*λ*_p_) and signal (*λ*_s_) beams to generate several nonlinear emissions (SHG_2*ωp*_, SFG, SHG_2*ωs*_). Measured intensity of the three nonlinear emissions generated by metasurface (colored lines), corresponding to SHG_2*ωp*_, SFG, and SHG_2*ωs*_ as a function of wavelength. The energy level diagram corresponding to the SFG nonlinear process is shown in the inset. Fourier plane images of nonlinear emissions generated by the GaP metasurface when illuminated by different polarization angles of the excitation beams. The orange and red arrows indicate the incident polarization of the pump and signal beams, respectively. The dashed white circle indicates the maximum angular field of view due to the numerical aperture of the collecting objective lens (NA = 0.9). The squares delimit the different SFG diffraction orders, and the white ones highlight the SFG diffraction orders of high intensity. Reproduced with permission [121]. Copyright 2022, American Chemical Society.

Based on the rapid progress of SHG, nondegenerated second-order effects such as sum frequency or differential frequency generation can be examined. Sum-frequency generation (SFG) is a nonlinear optical process where two photons, typically at different frequencies, interact within a material to produce a third photon whose frequency is the sum of the original two. Camacho-Morales et al. demonstrate efficient nonlinear sum-frequency generation (SFG) in multi-resonant GaP metasurfaces based on guided-wave BIC resonances [121]. In contrast to harmonic generation, the SFG process is enhanced when using nonparallel polarized input beams. The excitation of the metasurface by two near-infrared input beams generates strong SFG in the visible spectrum with a conversion efficiency of 2.5 × 10^−4^ W^−1^, two orders of magnitude higher than the one reported in Mie-type resonant metasurfaces. In addition, the nontrivial polarization dependence on the SFG process was verified, as shown in Figure 11.

#### 4.3.3 Spontaneous parametric down-conversion

**Figure 12:**
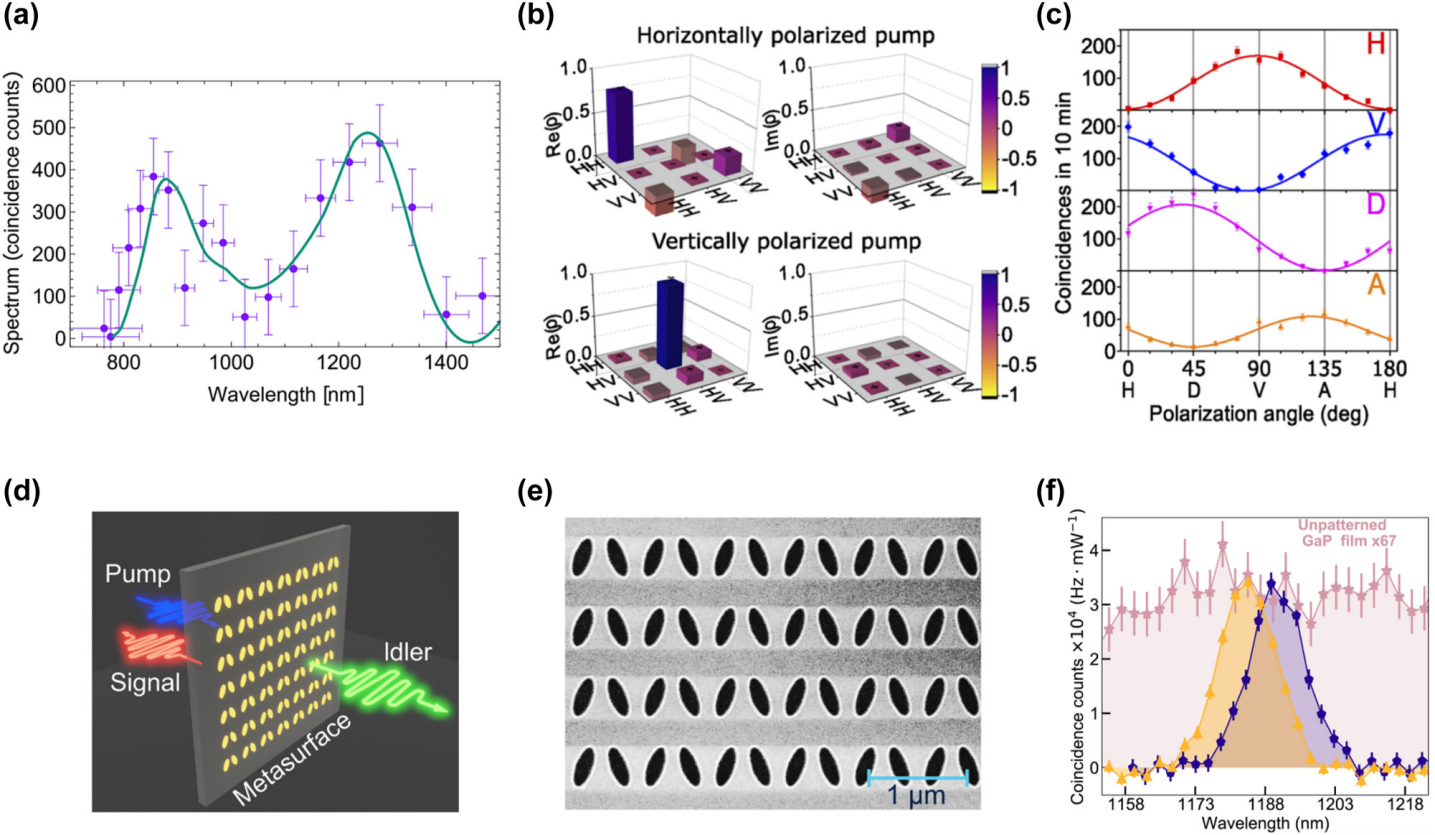
Spontaneous parametric down conversion using GaP devices. (a) SPDC spectrum as measured in GaP when pumped at 515 nm 100 µW. (b) Real and imaginary parts of the density matrix *ρ* of the photon pairs and (c) the number of coincidences versus the linear polarization angle selected. (d) SPDC in a metasurface with the signal and idler photons emitted in opposite directions. (e) A SEM view of the metasurface. (f) Enhancement of photon pair generation. (a) Reproduced with permission [55]. Copyright 2021, Optical Society of America. (b)–(c) Reproduced with permission [56]. Copyright 2022, Optical Society of America. (d)–(f) Reproduced with permission [80]. Copyright 2023, Royal Society of Chemistry.

Spontaneous parametric down-conversion (SPDC) is a well-developed tool using *χ* ^(2)^ from one photon to produce entangled photon pairs for practical applications, such as quantum imaging [122], [123], quantum key distribution [124], [125], and quantum metrology [126], as well as for tests of quantum mechanics [127]. Okoth et al. reported the observation of SPDC free of phase matching (momentum conservation) using a 6 μm thick layer of lithium niobate, revealing such processes at the microscale and nanoscale [128]. The ultrasmall thickness leads to a frequency spectrum an order of magnitude broader than that of phase-matched SPDC, while the strong two-photon correlations are still preserved due to energy conservation. About 400 nm thick GaP films [55] were typically chosen to achieve photon pair generation with a high coincidence-to-accidental ratio shown in Figure 12(a), due to a Fabry–Perot (FP) resonance. This FP cavity is tunable by the thickness of the GaP film and its underlying substrate, leading to enhanced optical near-fields concentrated inside the GaP at the required wavelength. By changing the pump polarization in Figure 12(b), the polarization state of photon pairs can be tuned from maximally entangled to almost disentangled on this GaP nanofilm, which is impossible in a single bulk source of SPDC [56]. Polarization entanglement, in combination with the broadband frequency spectrum, results in an ultranarrow Hong–Ou–Mandel effect of 12 fs and promises extensions to hyper-entanglement. The number of coincidences versus the linear polarization angle selected is shown in Figure 12(c). Generation of photon pairs via SPDC on metasurfaces was first demonstrated in lithium niobate [129]. With electric and magnetic Mie-like resonances at various wavelengths, the rate of pair production is enhanced up to 2 orders of magnitude, compared to an unpatterned film of the same thickness and material. Quasi-bound state in the continuum (BIC) resonances with high quality factor was adapted by gallium arsenic metasurfaces [130] to boost the quantum vacuum field and the emission of nondegenerate entangled photons within multiple narrow resonance bands and over a wide spectral range. A single resonance or several resonances in the same sample, pumped at multiple wavelengths, can generate multifrequency quantum states, including cluster states. This is also possible for GaP metasurface [80] supporting a quasi-bound state in the continuum; the schematic and SEM image of the metasurface can be seen in Figures 12(d) and (e), leading to remarkable emission directivities where the pair generation rate is enhanced 67 times compared to the case of an unpatterned film of the same thickness and material, which is illustrated in Figure 12(f). At the wavelength of the quasi-BIC resonance, photons are mostly emitted backward, while their partners, spectrally detuned by only 8 nm, are emitted forward.

Interestingly, quantum nanophotonic integrated circuits in thin-film InGaP has been demonstrated with a record-high ratio of 1.5 % between the single-photon nonlinear coupling rate (*g*/2π = 11.2 MHz) and cavity-photon loss rate, SPDC with an ultrahigh rate exceeding 27.5 MHz/µW-an order of magnitude improvement of the state of the art and a large coincidence-to-accidental ratio up to 1.4 × 10^4^ [120], showing In-doped GaP-OI as a potentially transcending platform for quantum nonlinear optics and quantum information applications. The SPDC performance of GaP devices is summarized in Table 9.

**Table 9:** SPDC using GaP devices.

Pump wavelength	Structure	Crystalline 100 or 111	*d*_14_ (pm/V)	Quality factor	Mechanism	Input power	Pair-generation rate		Year	Reference
685 nm	Film	100	N.A.	N.A.	SPDC	9 mW	0.20 ± 0.01 Hz	CW	2021	[55]
628 nm	Film	100	100	N.A.	SPDC	60 mW	N.A.	CW	2022	[56]
594 nm	Metasurface	100^a^	N.A.	100	SPDC	20 mW	3.4 mHz mW^−1^	CW	2023	[80]

^a^15° with respect to the normal to the wafer surface.

### 4.4 Third-order nonlinear optical nanodevices

#### 4.4.1 Third-harmonic generation

**Figure 13:**
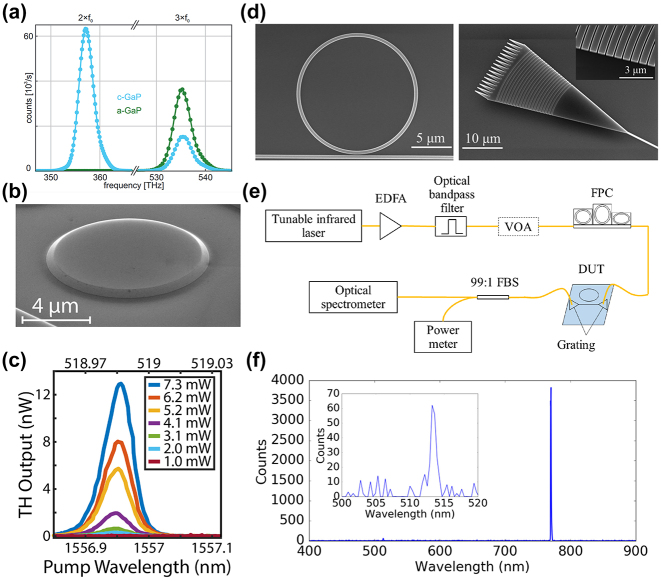
Third-harmonic generation using GaP devices. (a) Spectra of SHG and THG for the c-GaP (blue) and a-GaP (green) film for a pump frequency of *f*_0_ = 178.4 THz. Extracted values of *χ* ^(2)^ and *χ* ^(3)^ for the c-GaP (blue) and a-GaP (green) film as well as the bulk GaP crystal (orange dashed line). (b) SEM of a GaP microdisk before being undercut. (c) Third-harmonic output signal power for several pump wavelengths and input powers. (d) SEM images of fabricated photonic devices. Ring resonator comprising a 400 nm-wide circular waveguide with a radius of 7.5 μm and the associated 400 nm-wide bus waveguide. The gap between the ring and the bus waveguide is 160 nm. (e) Grating coupler at 30° tilt angle. Apparatus used for transmission measurements. (f) Spectrum of the diffracted light collected with the output fiber positioned above a ring resonator with 3.7 mW entering the device and an integration time of 5 s. The peak at 770 nm is the SHG signal. At 513 nm (inset), the THG signal is observed. (a) Reproduced with permission [8]. Copyright 2023, Wiley. (b)–(c) Reproduced with permission [82]. Copyright 2022, Optical Society of America. (d)–(f) Reproduced with permission [131].

Third-harmonic generation (THG) is a nonlinear optical process where three photons of the same frequency combine to form a single photon with triple the frequency (and one-third the wavelength) of the original photons. For efficient THG, the phase velocity of the interacting waves must be matched, often requiring careful control of the propagation medium and the geometry of the interaction. This limitation has been vastly relaxed at the nanoscale. Tilmann et al. reported that the third-order nonlinear susceptibility of amorphous GaP thin films is extracted from third-harmonic generation to be more than one order of magnitude larger than that of the crystalline material as shown in Figure 13(a), and generation of up to the fifth harmonic is observed [8]. McLaughlin et al. demonstrated the simultaneous generation of SHG and THG signals from a telecom wavelength pump in a GaP microdisk, which can be seen in Figure 13(b) [82]. THG signal was found to originate from both direct and cascaded sum frequency generation processes in Figure 13(c). Despite the relatively high material absorption in GaP at the third-harmonic wavelength, both of these processes can be significant, with relative magnitudes that depend closely on the detuning between the second-harmonic wavelengths of the cavity modes. Furthermore, Schneider et al. demonstrated that ring resonators with waveguide excitation have achieved an optical quality factor of 20,000 as well as a third-harmonic generation is observed at around 513 nm as shown in Figure 13(f) with 3.7 mW entering the device and an integration time of 5 s [60]. The apparatus used for transmission measurements is presented in Figure 13(e).

#### 4.4.2 High harmonic generation

**Figure 14:**
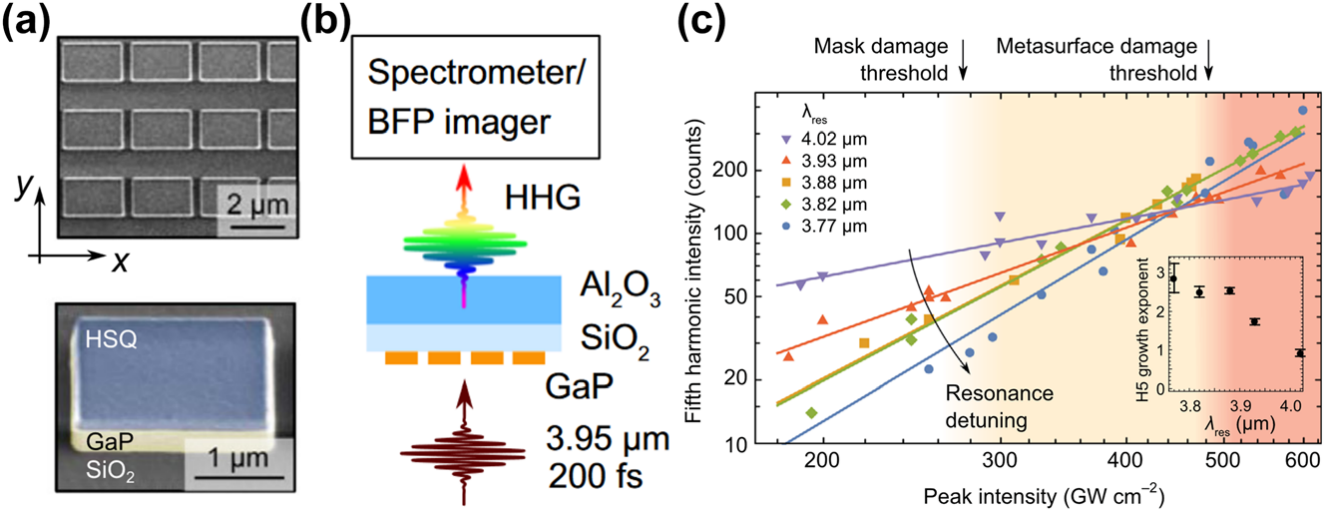
High-order harmonic generation using GaP devices. (a) Simplified schematic of the high harmonic generation (HHG) detection setup, with the detection arm represented by either a spectrometer or a back focal plane (BFP) imager. (b) Scanning electron microscope images, revealing the substrate (SiO_2_), the antenna material (GaP), and the lithography mask (hydrogen silsesquioxane, HSQ). (c) Zeroth diffraction order intensity of the H5 as a function of MIR pump intensity for five different metasurfaces with resonances at *λ*_res_, from the farthest from (blue circles) to the closest to (purple triangles) the driver wavelength. Solid lines: best fits to the power law ^(5)^ = *aI* ^*b*^. Deviation from the expected ^(5)^ = *I* ^5^ indicates the saturation of nonlinear response. Inset: power exponent *b* versus resonance wavelength *λ*_res_. The mask damage threshold and the metasurface damage threshold are shown for the most resonant metasurface *λ*_res_ = $\lambda_{res}^{\left(0\right)}$ = *λ*. (a)–(c) Adapted with permission from. (a)–(c) Reproduced with permission [57]. Copyright 2021, nature Publishing Group.

High harmonic generation (HHG) in GaP is a more complex and advanced nonlinear optical process compared to THG, which involves the generation of harmonics that are many times the frequency of the original incident light, either pumping from mid-infrared or reaching into the extreme ultraviolet (XUV) range. Shcherbakov et al. demonstrated an ultra-thin resonant GaP metasurface for highly efficient HHG driven by intense mid-infrared laser pulses [57]. The simplified schematic of the high harmonic generation (HHG) detection setup is presented in Figure 14(a), and the SEM image of the sample is shown in Figure 14(b). The even and odd harmonics covering a wide range of photon energies were generated between 1.3 and 3 eV with minimal reabsorption by single-shot measurements that avoid material damage, and the result is illustrated in Figure 14(c). Zalogina et al. reported the observation of up to a 7th harmonic generated from a single subwavelength resonator made of AlGaAs material [132]. This process is enabled by careful engineering of the resonator geometry for supporting an optical mode associated with a quasi-BIC in the mid-infrared spectral range at around *λ* = 3.7 μm pump wavelength, excited with an azimuthally polarized tightly focused beam. HHG is used in a variety of high-end applications, including the generation of coherent XUV light, attosecond pulse generation, and in studies of ultrafast dynamics.

#### 4.4.3 Four-wave mixing

**Figure 15:**
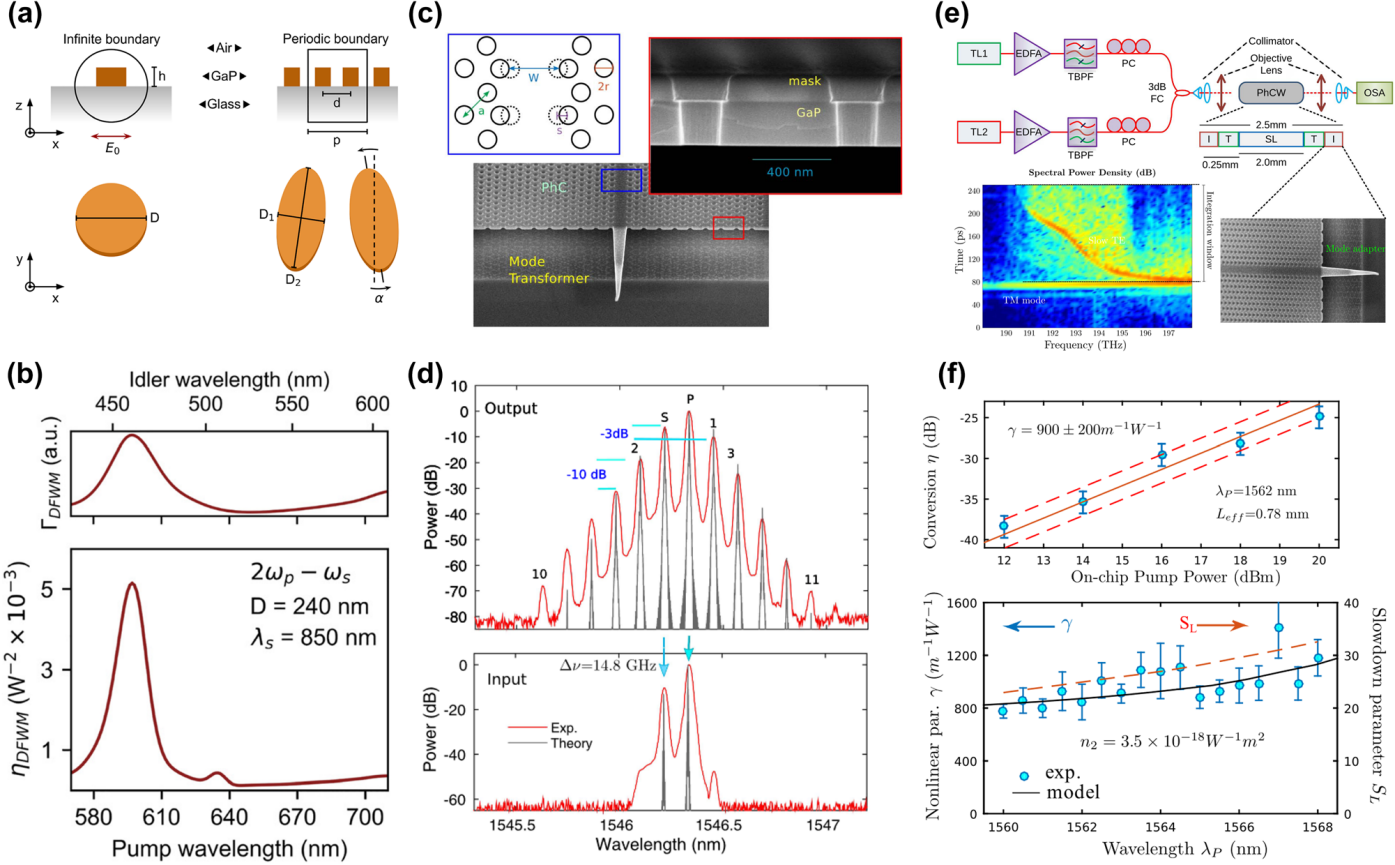
Four-wave mixing using GaP devices. (a) Schematic of the studied GaP platforms. Left: Different views of the individual disk resonator, including all relevant geometrical parameters. For the linear and nonlinear studies, the nanostructures were excited by linearly polarized light along the *x*-axis. Perfectly matched layers were used to simulate a semi-infinite glass substrate and air surrounding it. Right: Same as A but for the metasurface. The bottom shows the unit cell of the periodic array. To describe an infinite lattice, periodic boundary conditions were defined in the lateral edges of the square unit cell. (b) DFWM conversion efficiency (bottom) and third-order coupling factor Γ_DFWM_ (top) as a function of pump wavelength, with signal wavelength at 850 nm (MD), for a nanodisk with *D* = 240 nm. The top axis displays the output idler wavelength. (c) SEM image of a PhC waveguide made of a GaP slab and close-up before the removal of the etching mask (red rectangle). (d) Cascaded four-wave mixing experiment with ns pulses. Output spectra and raw conversion efficiency (*η*_*L*_) as a function of the peak power. The dashed line corresponds to *η* ∝ *P* ^2^. Detail of the output and the input at maximum peak power: experimental (red) and calculated (black) spectra. (e) Experimental setup for the measurement of the FWM (TL, tunable laser; EDFA, erbium-doped fiber amplifier; TBPF, tunable bandpass filter; PC, polarization controller; FC, fiber coupler; OSA, optical spectrum analyzer) PhC waveguide sections. Inset, PhC waveguide sections with input (I), transition (T), and slow-light (SL) sections. The density plot of the spectral power density transmitted depending on the propagation delay; the dynamic range of the logarithmic map is 70 dB. SEM image of the input section (mode adapter) of the waveguide. (f) Conversion efficiency (symbols) versus the on-chip pump power and fit (solid line) and confidence intervals (dashed). Extracted nonlinear parameter *γ* as a function of the pump wavelength (symbols) compared with the model (dashed line) and the slow-down scaling *S*_*L*_ = $n_{g}^{2}/n^{2}$ . (a)–(b) Reproduced with permission [115]. Copyright 2021, De Gruyter. (c)–(d) Reproduced with permission [103]. Copyright 2018, Optical Society of America. (e)–(f) Reproduced with permission [107]. Copyright 2019, Optical Society of America.

Four-wave mixing (FWM) is another nonlinear optical process. In FWM, three light waves interact within a nonlinear material to generate a fourth wave. The process can occur in several forms, such as degenerate and nondegenerate FWM, depending on the frequencies of the input waves. Moretti et al. numerically explored degenerate four-wave mixing, demonstrating giant per unit cell conversion efficiencies of up to ∼2 W^−1^ and ∼60 W^−2^, respectively, when considering realistic introduced asymmetries in the metasurface [115]. Figure 15(a) shows such configuration outperforms by up to more than four orders of magnitude, the response of low quality factor Mie, or anapole resonances in individual GaP nanoantennas with engineered nonlinear mode-matching conditions. DFWM conversion efficiency (bottom) and third-order coupling factor Γ_DFWM_ (top) as a function of pump wavelength are illustrated in Figure 15(b). Martin and his colleagues applied peak power intensity levels of 50 GW/cm^2^ safely reaching in a suspended GaP membrane [103]. SEM image of a PhC waveguide made of a GaP slab and close-up before the removal of the etching mask (red rectangle) is presented in Figure 15(c). Consequently, the field enhancement is exploited to a far greater extent to achieve efficient and strong light–matter interaction, as shown in Figure 15(d). As an example, parametric interactions are shown to reach a deeply nonlinear regime, revealing cascaded four-wave mixing leading to comb generation and high-order soliton dynamics. Xie et al. measured and extracted nonlinear coefficient ranges from about 800 W^−1^ m^−1^ to 1400 W^−1^ m^−1^ through CW four-wave mixing, consistently with an estimated material nonlinearity *n*_2_ = 3.5 × 10^−18^ W^−1^ m^2^ [107]. The experimental setup for the measurement of the FWM is illustrated in Figure 15(e). The nonlinear parametric coefficient through FWM is shown in Figure 15(f) with detunings large enough (>100 GHz) to rule out the contributions from slower effects.

#### 4.4.4 All-optical switching using the Kerr effect

Optical Kerr effect (OKE) is also a third-order nonlinear optical phenomenon where the refractive index of a material changes in response to the intensity of an applied light field. The refractive index *n* of the material becomes dependent on the intensity *I* of the applied light, following the relation *n* = *n*_0_ + *n*_2_ *I*, where *n*_0_ is the linear refractive index and *n*_2_ is the nonlinear index coefficient. OKE is a spontaneous ultrafast process, occurring on the order of femtoseconds. This makes itself suitable for high-speed applications in photonics, such as ultrafast optical switching. At high intensities, the optical Kerr effect in materials such as GaAs may be accompanied by thermal effects due to linear and nonlinear photon absorption, which needs to be seriously considered, especially in applications involving CW laser beams.

**Figure 16:**
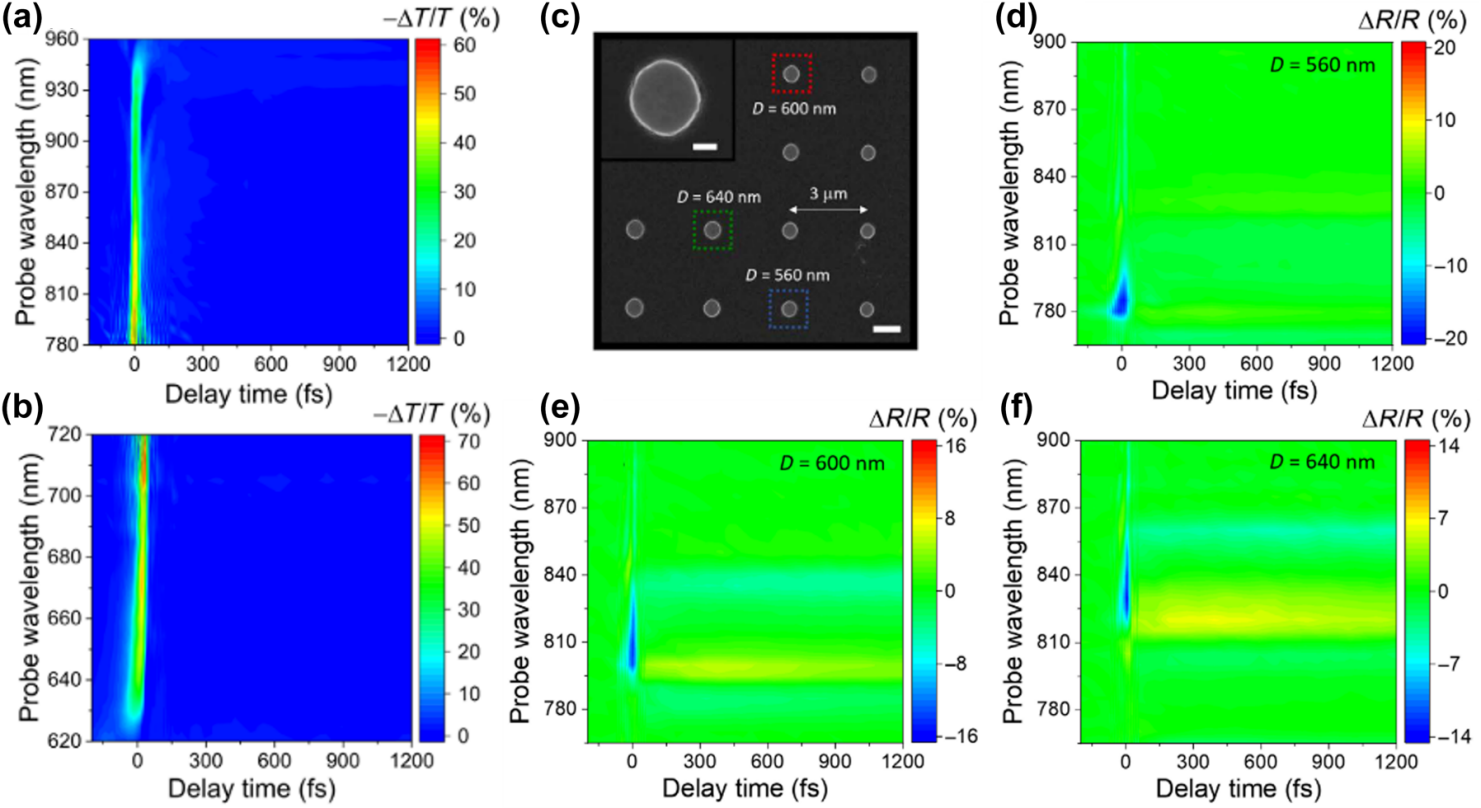
All-optical switching using GaP devices. (a) Differential transmissivity spectra of the GaP sample as a function of pump–probe delay time, when pumped with the short-wavelength beam. (b) Differential transmissivity spectra of the GaP sample as a function of pump–probe delay time, when pumped with the long-wavelength beam. (c) Scanning electron microscopy image of the fabricated sample. The inset shows a magnified view of a nanodisk of 600 nm diameter. Scale bars, 1 μm (main image) and 200 nm (inset). (d–f) Differential reflectivity spectra of individual nanoantennas of diameters *D* = 560, 600, and 640 nm, registered by pumping the sample at *P* = 10 pJ/μm^2^, with a 5:1 pump–probe fluence ratio. (a)–(b) Reproduced with permission [70]. Copyright 2019, American Association for the Advancement of Science. (c)–(f) Reproduced with permission [54]. Copyright 2020, American Association for the Advancement of Science.

GaP is one of the few available materials with strong optical nonlinearity and negligible losses in the visible (*l* > 450 nm) and near-infrared regime. Grinblat et al. demonstrated that a GaP film could generate sub-30-fs (full width at half maximum) transmission modulation of up to ∼70 % in the 600- to 1,000-nm wavelength range [70]. The differential transmissivity spectra of the GaP sample as a function of pump–probe delay time, when pumped with the short- and long-wavelength beams, are illustrated in Figure 16(a) and (b), respectively. This is supported by nonlinear simulations using parameters measured by the Z-scan approach, indicating that the transmission modulation arises from the optical Kerr effect and two-photon absorption. No slower free-carrier contribution is detected due to the absence of linear absorption, leading to all-optical switching at modulation speeds of up to 20 THz. Grinblat et al. further demonstrated that a crystalline GaP nanodisk could yield differential reflectivity modulations of up to −40 %, with characteristic modulation times between 14 and 66 fs, when probed at the anapole excitation (AE) [54]. The scanning electron microscopy image of the fabricated sample can be seen in Figure 16(c). Figure 16(d)–(f) show the differential reflectivity spectra of individual nanoantennas of diameters *D* = 560, 600, and 640 nm, respectively. The ultrafast all-optical modulation through both the optical Kerr effect (OKE) and two-photon absorption (TPA) in the visible/near-infrared range highly outperforms previous reports on sub-100-fs all-optical switching from resonant nanoscale dielectrics, which have demonstrated modulation depths no larger than 0.5 %.

#### 4.4.5 Phonon-assisted Raman scattering

Raman scattering occurs when light interacts with vibrational or rotational energy levels within a molecule or a crystal lattice. The energy of the scattered light is shifted from the incident light by the energy associated with the vibrational or rotational transitions. This shift can be either higher (blue-shift) or lower (red-shift) in energy (Stokes and anti-Stokes scattering).

**Figure 17:**
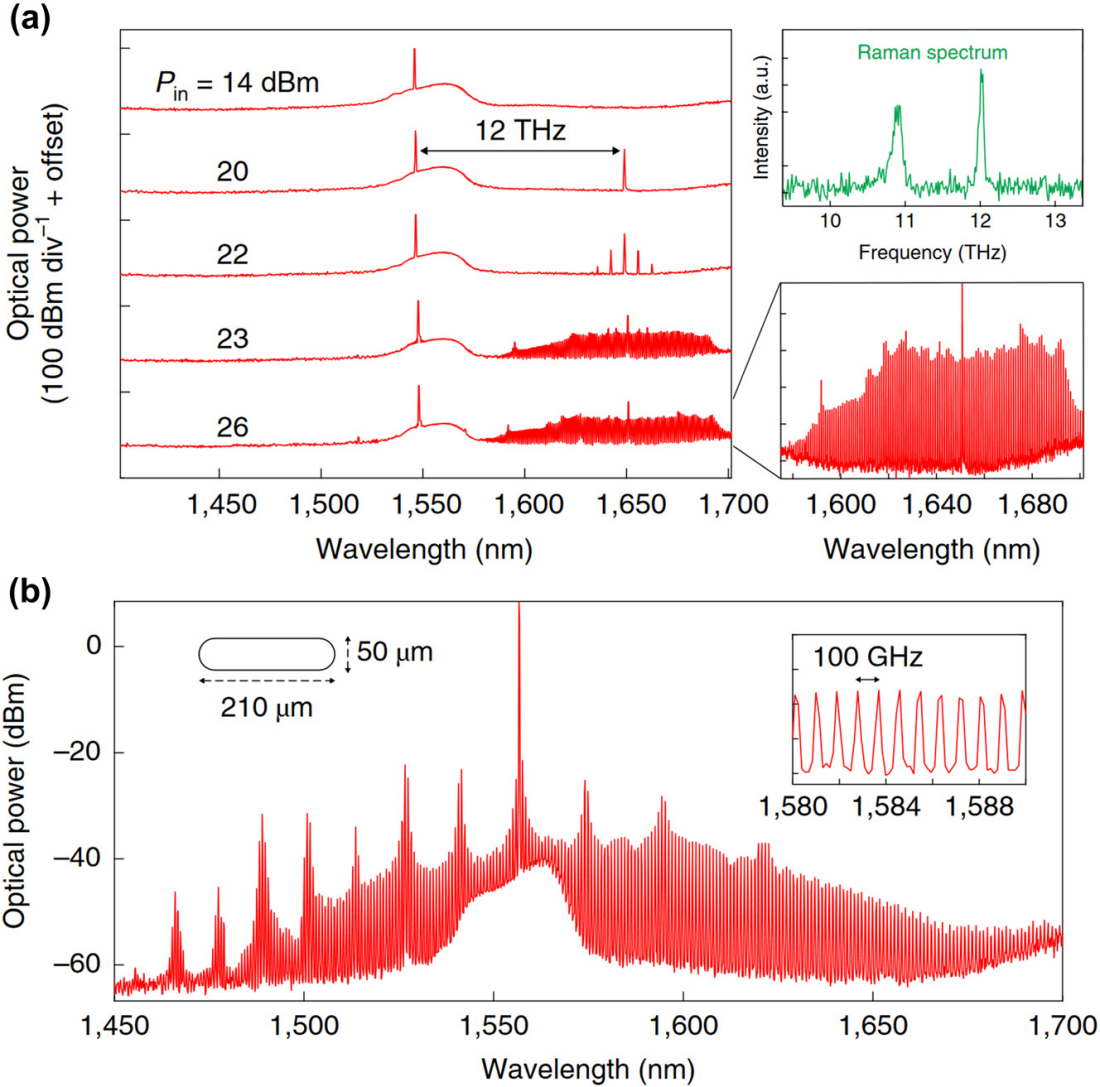
Phonon-assisted Raman or Brillouin scattering using GaP devices. (a) Broadband frequency comb generated in a cladded racetrack resonator with FSR of 100 GHz, using *P*_in_ ≈ 26 dBm. (b) Raman-shifted comb observed on a different mode of the same device [16]. (a)–(b) Reproduced with permission [16].

Stimulated Raman scattering (SRS) competes with FWM in waveguides with normal or weak dispersion, which plays an important role in GaP waveguide resonators cladded with silica. This effect reduces *D*_2_ by an order of magnitude and displaces the center of the anomalous window to ∼1,650 nm. Wilson et al. showed FWM and SRS in a 100 GHz racetrack resonator with *Q* ≈ 2 × 10^5^ (Figure 17(a)) [16]. For a subset of modes, broadband (>200 nm) frequency combs centered at the pump wavelength are generated by FWM. For others, FWM is preceded by efficient Raman lasing (20 dBm threshold, 10 % conversion efficiency) with a Stokes frequency of 12 THz. The selectivity between FWM and SRS, and the absence of SRS in resonators with smaller radii, is probably due to the narrowness of the Raman transition (inherent to crystalline material) and is possibly tunable via crystal orientation or optical polarization 40. Independent Raman spectroscopy of the GaP growth substrate (Figure 17(b)) confirms that the 12 THz transition is less than 100 GHz wide.

#### 4.4.6 Pulse compression and spectrum broadening

**Figure 18:**
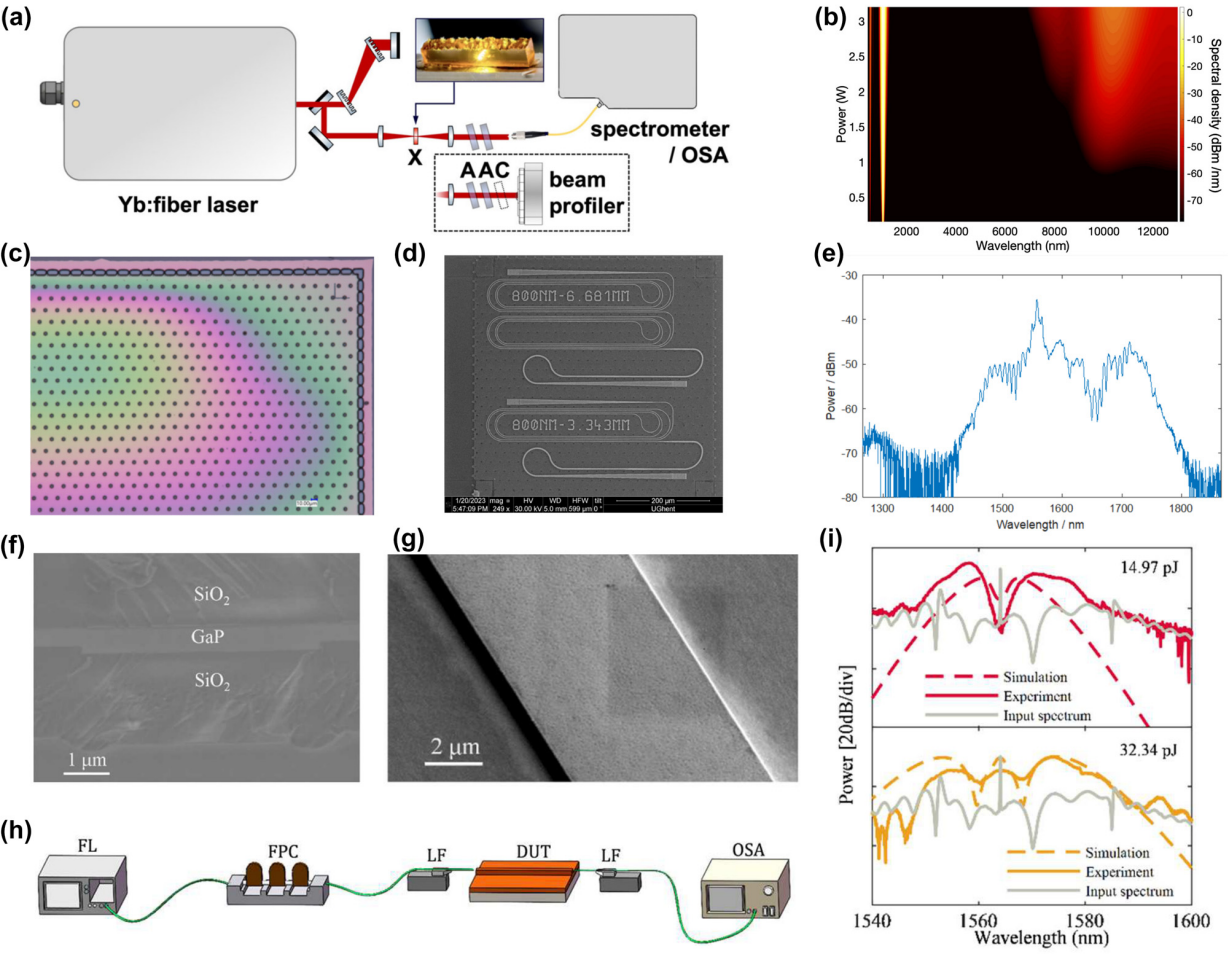
Supercontinuum and soliton generation using GaP devices. (a) Supercontinuum generation experiment. Stretched pulses from a Yb: fiber laser were de-chirped in a grating compressor before being focused into an OPGaP crystal (X). The resulting supercontinuum was measured using a visible spectrometer and optical spectrum analyzer (OSA), as well as a beam profiling camera. (b) Intense pump light was rejected using two attenuators (A), and beam profiling employed different color filters (C) to isolate: pump light at 1,040 nm; full-spectrum NEE simulation, showing long-wave infrared generation above 7 μm, corresponding to idler radiation from difference frequency mixing between 520 nm and wavelengths shorter than 562 nm. (c) An optical microscope picture of a suspended coupon still attached to the source wafer. (d) SEM picture of one of the 520 × 520 μm printed coupons with two spirals. (e) Measured supercontinuum spectrum of a GaP waveguide with dimensions *l* = 13 mm, *w* = 750 nm, and *h* = 300 nm. (f) The SEM image of a GaP rib waveguide after stripping photoresist. (g) The SEM image of the cross section of the inverted GaP-OI rib waveguide. (h) The schematic of the spectrum broadening experiment setup. FL, femtosecond laser; FPC, fiber polarization controller; LF, lensed fiber; DUT, device under test; OSA, optical spectrum analyzer. (i) Measured and simulated output spectra at different pulse energies from the waveguide with a width of 1.8 μm. (a)–(b) Reproduced with permission [69]. Copyright 2020, Optical Society of America. (c)–(e) Reproduced with permission [22]. Copyright 2023, Optical Society of America. (f)–(i) Reproduced with permission [13]. Copyright 2023, Optical Society of America.

GaP possesses a conspicuous *χ* ^(3)^ coefficient that can facilitate the occurrence of third-order nonlinear phenomena. Rutkauskas et al. introduced a new mechanism combining second- and third-order nonlinearities to produce broadband visible light in orientation-patterned gallium phosphide (OP-GaP) [69]. A supercontinuum from the blue/green to the red shown in Figure 18(a) and (b) is produced from 32 nJ 1,040 nm fs pulses, and a nonlinear-envelope-equation model including *χ* ^(2)^ and *χ* ^(3)^ nonlinearities implies that high-order parametric gain pumped by the second-harmonic light of the laser and seeded byself-phase-modulated sidebands is responsible. Reis et al. used a spiral GaP waveguide (shown in Figure 18(c) and (d)) with a length of 13 mm and 750 × 300 nm^2^ cross section excited by 270 fs pulses at 1,550 nm to observe supercontinuum generation [22]. A peak power under 100 W inside the waveguide was sufficient to generate the spectrum shown in Figure 18(e). Cheng et al. demonstrated supercontinuum generation (SCG) from GaP-OI waveguides [13], [61]. The SEM image of a GaP rib waveguide after stripping photoresist of the cross section of the inverted GaP-OI rib waveguide is shown in Figures 18(f)–(g), respectively. Figure 18(h) shows the schematic of the spectrum-broadening experiment setup. The supercontinuum spectra exhibit characteristic mesa shapes with split peaks, indicating that the spectrum broadening is governed by a self-phase modulation (SPM) mechanism, which is illustrated in Figure 18(i). By correlating the nonlinear phase shift with the product of peak power and the effective length, the nonlinear refractive index of GaP material, *n*_2_, is extracted to be 1.9 × 10^−17^ m^2^/W, nearly two orders of magnitude larger than that of Si_3_N_4_ (∼0.2 × 10^−18^ m^2^/W [133]).

GaP’s alloys, such as InGaP, also excel in pulse compression and spectrum broadening. Colman et al. demonstrated the first experimental observations of optical solitons and pulse compression in ∼1-mm-long GaInP PhC waveguides [134]. Suppression of two-photon absorption in the GaInP material is crucial to these observations. The device is made of Ga_0.5_In_0.5_P in order to suppress the two-photon absorption in the Telecom spectral range considered here [135]. Compression of 3-ps pulses to a minimum duration of 580 fs with a simultaneously low pulse energy of ∼20 pJ is achieved. Moreover, Husko et al. demonstrate through both experiment and theory that nonlinear photocarrier generation can induce soliton fission [136]. The nonlinear spatial and temporal evolution of optical pulses can be directly observed *in situ* in a nanophotonic semiconductor waveguide using near-field measurements. The experiment exceeds the minimum threshold by an order of magnitude using the analytic formalism describing the free-carrier dispersion (FCD) perturbation.

#### 4.4.7 Kerr optical frequency comb generation

**Figure 19:**
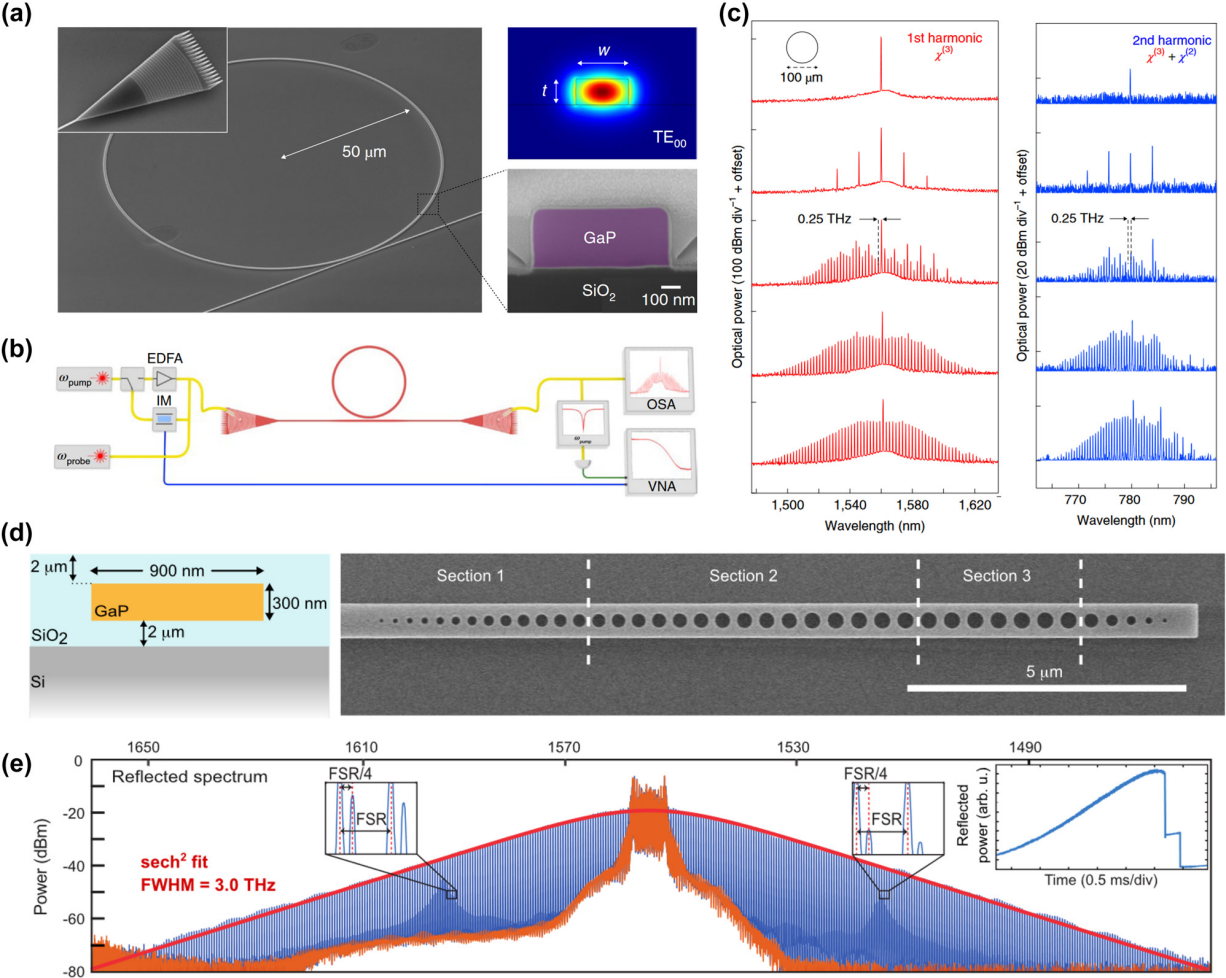
Optical Kerr comb and soliton generation using GaP devices. (a) Scanning electron microscopy (SEM) image of an uncladded ring resonator (50 μm radius) with integrated bus waveguide and grating couplers (inset). The waveguide thickness and width are 300 nm and 500 nm, respectively. Finite element simulation of the TE_00_ waveguide mode. SEM image of the waveguide in cross section. (b) Set-up for pump–probe response and frequency comb measurements. EDFA, erbium-doped fiber amplifier; IM, intensity modulator; OSA, optical spectrum analyzer; VNA, vector network analyzer. (c) Frequency comb generation in an uncladded ring resonator with a radius of 50 μm. From top to bottom, the laser power is fixed, and the laser-cavity detuning is incrementally reduced. (d) Schematic of the GaP waveguide cross section with dimensions (not drawn to scale). Scanning electron microscopy image of a fabricated PCR. (e) Spectra measured in reflection of the soliton state (blue) and off-resonance (orange) for an average input power in the bus waveguide of ∼70 mW. The two insets above the spectra show the positions of the additional weak lines offset from the main soliton comb lines. The inset on the right is an oscilloscope trace recorded as the laser is scanned through resonance [24]. (a)–(c) Reproduced with permission [16]. Copyright 2020, nature research. (d)–(e) Reproduced with permission.

The strong third-order nonlinearity can promote Kerr optical frequency comb (OFC) generation in GaP resonators, first observed in GaP-OI resonators by researchers from IBM Zurich [16]. Those resonators were fabricated on GaP on oxide heterogeneous wafers that were molecule-bonded by leveraging the assistance of thin Al_2_O_3_ layers, where the SEM image of the device is shown in Figure 19(a). The waveguide cross section of 500 nm × 300 nm (width × height) supports anomalous dispersion and OFC generation around 1,560 nm. Figure 19(b) shows the set-up for pump–probe response and frequency comb measurements. The *Q* factor of the resonator is as high as 3 × 10^5^, corresponding to a propagation loss of 1.2 dB/cm, which enables low-threshold parametric oscillation at merely 3 mW, illustrated in Figure 19(c). Besides Kerr OFC generation at 1,560 nm, frequency-doubled OFCs around 780 nm were also experimentally observed thanks to the *χ* ^(2)^ nonlinearity in GaP. The free spectrum range (FSR) of the OFC around 780 nm is the same as that around 1,560 nm, indicating that sum frequency generation (SFG) plays a dominant role during the frequency translation process. Unfortunately, not a single soliton OFC was reported on these GaP-OI ring resonators, partially due to the non-negligible thermo-optic coefficient (TOC) that hinders the capture of the soliton state. This challenge was later addressed by the same team using pulsed pumping [24]. They used an electro-optic (EO) comb whose repetition rate is 1/4 of the FSR of the GaP-OI cavity to pump the Kerr soliton. This method can reduce the thermal effects compared to CW operation at the same peak power level. The GaP Fabry–Perot cavity comprises a straight waveguide whose cross section is 900 nm × 300 nm in the middle and two PhC reflectors (PCR) whose cross sections are 700 nm × 300 nm with a series of holes at two ends. The schematic of the GaP waveguide cross section and the SEM image of a fabricated PCR is presented in Figure 19(d). The normal dispersion of the straight waveguide is compensated by the chirped PCRs. Due to the large refractive index of GaP, chirped PCRs can be realized by only 15-unit cells while simultaneously achieving a high reflectivity over about 20 THz and an intrinsic quality factor for a GaP resonator of 1.2 × 10^6^. A single soliton around 1,550 nm can be generated with an average input power as low as 23.6 mW, as illustrated in Figure 19(e). The low-power operation is enabled by the record-low waveguide propagation loss of 0.85 dB/cm.

Fueled by experimental progress, theoretical investigations on GaP Kerr microcombs advance rapidly. For microcombs that rely on *f* − 2*f* self-referencing to stabilize the frequencies, it is desirable to have a wide spectral bandwidth. It has been pointed out that a flat anomalous dispersion profile is in favor of a broadband OFC generation [137]. To flatten the dispersion profile, Geng et al. proposed a concentric GaP double ring resonator structure [138]. The fully separated inner and outer rings enable avoided mode crossing, which further ignites the symmetric mode and the antisymmetric mode. The anomalous dispersion of the antisymmetric mode can be fine-tuned by the double-ring geometric parameters, including waveguide widths, gap, radius, etc., to facilitate broadband OFCs. Ji et al. further improved the GaP concentric ring resonators by adding a tuning knob of the gap depth [139]. With the help of the partially etched gap, an unprecedently broad anomalous dispersion region spanning from short wave-infrared to mid-infrared can be obtained. The anomalous dispersion is enabled by two mechanisms: avoided mode crossing at shorter wavelengths and high-order mode at longer wavelengths. Thus, such a resonator theoretically supports two-color Kerr OFCs around 1,550 nm and 3,100 nm. It is worth noting that two-color OFCs can also be generated through cascaded *χ* ^(3)^ and *χ* ^(2)^ nonlinearities. Researchers from the Chinese Academy of Sciences investigated the mid-infrared and near-infrared frequency comb generation in GaP microrings followed by a straight waveguide segment [140]. Mid-infrared Kerr OFCs are generated inside the GaP microrings, while the near-infrared second-harmonic (SH) OFCs are frequency doubled by the strip waveguide. Actually, such sequential processes can coincide inside the microresonators, given the optimized waveguide geometries to meet the phase-matching conditions [16]. Wu et al. specifically investigated the generation and tunability of *χ* ^(2)^-translated OFCs in GaP-OI resonators [141]. A detailed geometry optimization approach was given to satisfy the anomalous dispersion and cyclic phase matching conditions synergistically. On the optimized structure, an intracavity SHG efficiency as high as 71.5 %/W was predicted. Furthermore, tuning the OFC spectral shape is possible by post-trimming the waveguide geometry or tweaking the device temperature. Nikolai et al. have demonstrated the ability to amplify ultra-weak signals using low-loss photonic integrated waveguides on an insulator with gallium phosphide, centered around a wavelength of 1,550 nm. They achieved a parameter gain of up to 35 dB, extending over six orders of magnitude of input power, covering optical frequency combs and communication signals [142]. The third-order nonlinear effects using GaP devices are summarized in Table 10.

#### 4.4.8 Optical parametric oscillation

One photonic device in particular that scientists have been trying to miniaturize and put onto a chip is the optical parametric oscillator (OPO), a source of light where two beams, named the signal and idler, are generated from a pump beam by nonlinear optics. While this is usually a second-order nonlinear effect (using two beams to generate a new one), it can also be adapted for third-order nonlinearity. Marty et al. reported a 20-μm-long GaP PhC cavity operating at telecom wavelengths [143]. Parametric oscillation is reached when high quality factor modes are thermally tuned into a triply resonant configuration, whereas any other parametric interaction is strongly suppressed. The lowest pump power threshold is estimated to be 50–70 μW, close to the lowest value ever reported for a microring OPO (36 μW) [144]. Chip-scale OPOs are desirable as the signal and idler are correlated and could yield an integrated light source for generating entangled photon pairs for applications in integrated quantum optics. This source behaves as an ideal degenerate optical parametric oscillator, addressing the needs in the field of quantum optical circuits and paving the way toward the dense integration of highly efficient nonlinear sources of squeezed light or entangled photons pairs.

### 4.5 Optoelectrical devices

#### 4.5.1 Photodetectors

GaP photodetectors, as semiconductor devices, can convert photons whose wavelengths are shorter than 550 nm into electrons. They become essential, particularly when combining the UV regime as well as the blue portion of the visible spectrum.

**Figure 20:**
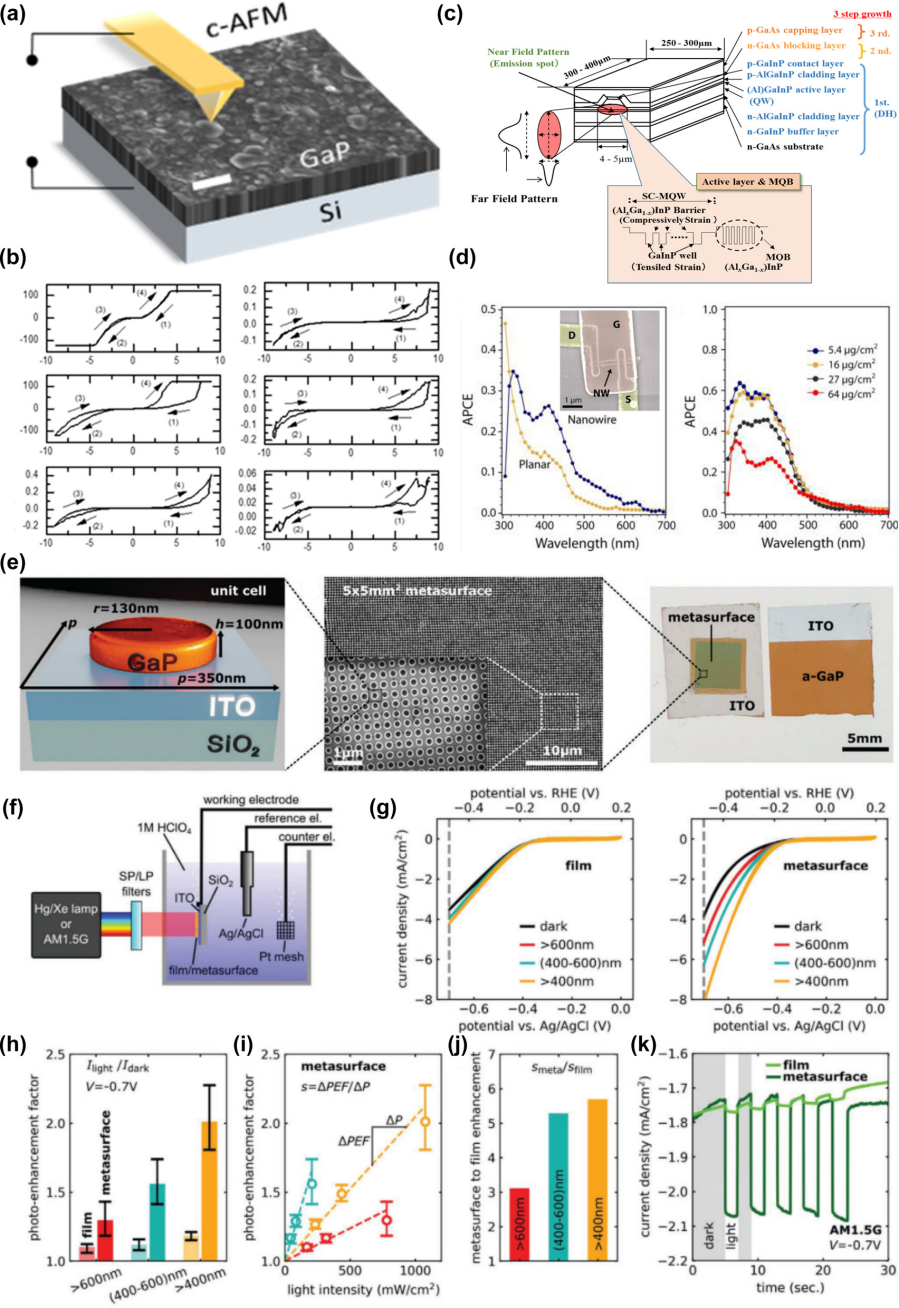
Optoelectrical GaP devices. (a) Scanning electron microscopy (SEM) image of the GaP film grown on a Si substrate and a schematic illustration of the cAFM setup used to measure the resistive switching performance at the nanoscale. (b) Current–voltage (I–V) curves measured under different wavelengths of light irradiation. The incident photon energy for each I–V loop from top to bottom panel is 2.95, 2.75, 2.64, 2.38, 2.25, and 2.17 eV. (c) The 630-nm band AlGaInP laser diode. (d) APCE spectra of GaP nanowire photocathodes at different loading amounts. In both figures, the GaP nanowires were doped with 0.98 atom % Zn versus Ga, and the spectra were measured at 0.1 V versus RHE under simulated one-sun conditions (AM1.5G). Inset: SEM image of a single-nanowire FET device. (e) Scheme of the metasurface unit cell. SEM image with different zooms into the large-scale nanostructure. Photograph of the photoelectrodes with the imprinted metasurface on the left and a 100 nm thick continuous a-GaP film, both on top of a 100 nm thick layer of ITO. (f) Experimental setup for the PEC characterization of the a-GaP film and metasurface. (g) Cyclic voltammetry on the photoelectrodes under dark conditions and red, green, and white light illumination. (h) Photo-enhancement factor (photo divided by dark currents) for the three different wavelength ranges at *V* = −0.7 V versus Ag/AgCl. (i) Linear power dependence of the photo-enhancement factor, with *s* representing the slope for each of the three investigated illumination conditions. (j) Metasurface-to-film enhancement factors calculated from the data in (i) as the ratio between the slopes of power-dependent photo-enhancement factors from metasurfaces and continuous films. (k) Chopped-light chronoamperometry curves for an a-GaP metasurface compared to continuous a-GaP film at a potential of −0.7 V versus Ag/AgCl under simulated AM 1.5G 1 Sun illumination powers. (a)–(b) Reproduced with permission [77]. Copyright 2021, American Chemical Society. (c) Reproduced with permission [144]. Copyright 2017, Molecular Diversity Preservation International. (d) Reproduced with permission [49]. Copyright 2012, American Chemical Society. (e)–(k) Reproduced with permission [64]. Copyright 2021, Wiley.

Novel functions can be added to conventional GaP photodetectors. Kurnia et al. investigated nanoscale resistive switching characteristics of GaP thin films that were directly grown on Si as a function of incident light [145]. The presence of point defects and structural disorder accelerated the formation of conductive channels along the grain boundaries (GB). Current–voltage sweeps were obtained from a representative GB region for each specific photon energy, as shown in Figure 20(a) and (b). It is interesting to note that the switching current changes significantly as a function of the photon energy. Photoconductive atomic force microscopy (phAFM) measurement confirmed that photocurrents could be harvested below the band gap due to the presence of mid-gap electronic states. These states can effectively reduce the energy required for photoconduction. phAFM measurements show (i) a systematic increase in surface conduction as a function of increasing photon energy and (ii) photoactivity at sub-bandgap energies, confirming the role of mid-gap states. These results show that a GaP film directly grown on Si is a promising candidate material for nonvolatile resistive switching memory and nanophotonic applications.

#### 4.5.2 Quantum-well laser diodes

A thin layer of semiconductor materials (the “well”) can be sandwiched between layers of another semiconductor creating a potential well where electrons and holes are confined in the dimension perpendicular to the layers. This confinement leads to quantized energy states for the electrons and holes in the well, which enhances the efficiency of the electron–hole recombination process necessary for laser action. Hamada et al. fabricated highly ordered gallium indium phosphide (GaInP) and AlGaInP layers grown on the (100) GaAs substrates (shown in Figure 20(c)), where the bandgap at 300 K is 1.791 eV [145]. The transverse stabilized AlGaInP with strain-compensated quantum well structure performed reasonably well as laser diodes by controlling the misorientation angle and directions of GaAs substrates. The same structure is also applied to quantum dots laser diodes.

#### 4.5.3 Photoelectrodes

Broadband solar light harvesting plays a crucial role in efficient energy conversion. Tailoring optical properties in photocatalysts by nanostructuring them can help increase solar light harvesting efficiencies in a wide range of materials.

On one hand, Liu et al. introduced P-type dopant zinc during synthesis to optimize the response of the GaP photocathode (shown in Figure 20(d)). The electrical properties of Zn-doped GaP nanowires were studied to confirm their p-type conductivity. After optimizing the nanowire diameter and Zn doping concentration, progress in the overall photoabsorptive photon flux conversion efficiency (APCE) was achieved. By removing the necessity of charge transport through the nanowire/nanowire interface, the submonolayer of Zn-doped GaP nanowires reaches 57 % efficiency at 400 nm (0.1 V versus RHE).

On the other hand, Hüttenhofer et al. presented a systematic study of coupling mechanisms in rectangular arrays of amorphous GaP nanodisks that support anapole excitations at 600 nm, which is within the lossy spectral regime of the material [90]. The experimental findings show that maximum visible light extinction by the array and maximum absorption in the GaP is not achieved by the densest packing of resonators. Counterintuitively, increasing the array periodicities such that collective effects spectrally overlap with the anapole excitation of a single particle leads to an absorption enhancement of up to 300 % compared to a single disk. Hüttenhofer et al. developed a design procedure for large-scale nanofabrication using imprint lithography for metasurface photoelectrodes [64]. Amorphous GaP is shown in Figure 20(e) as cost-effective, high sample throughput while retaining the precise signature of the engineered photonic states. Figure 20(f) shows the experimental setup for the PEC characterization of the a-GaP film and metasurface. Photoelectrochemical measurements under hydrogen evolution reaction conditions and sunlight illumination reveal the contributions of the respective resonances and demonstrate an overall photocurrent enhancement of 5.7, compared to a planar film, which is illustrated in Figure 20(h–k). These results provide a fundamental understanding of tailored light absorption in coupled anapole resonators and reveal important design guidelines for advanced metasurface approaches in a wide range of energy conversion applications.

### 4.6 Optomechanical and mechano-optical devices

**Figure 21:**
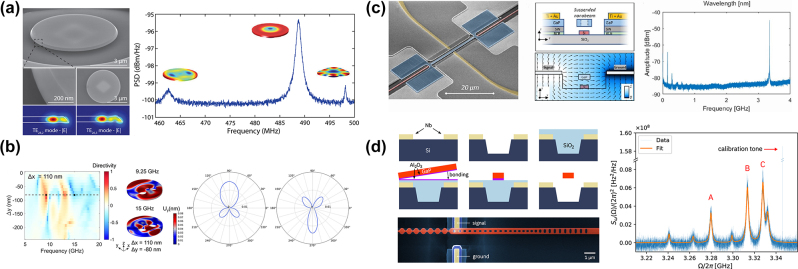
Optomechanical and mechano-optical GaP devices. (a) The SEM of microdisk and a radial breathing mode at 488 MHz. (b) The antenna emits vibration at two opposite directions at 9.25 GHz and at 15 GHz. (c) GaP PhC stimulated by Si waveguide and modulated by electro-field. Original peak at 3.35 GHz. (d) GaP PhC beam with Nb cathode and resulted 3 mechanical vibrational peaks, A at 3.173 GHz, B at 3.227 GHz and C at 3.249 GHz. (a) Reproduced with permission [81]. Copyright 2014, American Institute of Physics. (b) Reproduced with permission [146]. Copyright 2023, Royal Society of Chemistry. (c) Reproduced with permission [62]. Copyright 2023, American Chemical Society. (d) Reproduced with permission [58]. Copyright 2022, Nature Publishing Group.

Cavity optomechanics in GaP microdisks [81] was demonstrated a decade ago, with intrinsic quality factors >2.8 × 10^5^, as shown in Figure 21(a). Optomechanical coupling between optical modes of the microdisk around 1.5 μm and several mechanical resonances yield to an optical spring effect consistent with a theoretically predicted optomechanical coupling rate *g*_0_/2π roughly 30 kHz for the fundamental mechanical radial breathing mode at 488 MHz.

Yan et al. [146] theoretically showed that detectable mechanical vibrations ranging from gigahertz to terahertz in GaP nanoantennas are found to surpass their metallic counterparts in a 400 nm–800 nm pump–probe configuration. Pronounced directional propagation for launching surface acoustic waves (SAWs) with a double-hole structure rather than with a one-hole configuration using low-aspect ratio GaP disks illustrated in Figure 21(b) being probed near their optical anapole resonance.

The large electronic band gap and the resulting low optical absorption of GaP were utilized to demonstrate quantum behavior of a 2.9 GHz mechanical mode coupled to a high quality factor optical resonator in the telecom band [99], opening the way for realizing noise-free quantum transduction between microwaves and optics.

IBM Zurich designed and fabricated 1D GaP PhC cavities made with optical quality factors as high as 1.1 × 10^5^ at 200 THz and a co-localized mechanical mode with a frequency of 3 GHz [59]. The high vacuum optomechanical coupling rate (*g*_0_/2π = 400 kHz) permits amplification of the mechanical mode into the mechanical lasing regime with input power as low as 20 μW, where a multiphoton cooperativity of *C* > 1. A low phase noise electro-optomechanical oscillator directly working in the GHz frequency range was achieved on 1D photonics crystal GaP heterogeneously integrated on silicon-on-insulator circuitry [62] (shown in Figure 21(c)), exhibiting a short-term stability of 0.7 Hz linewidth and a long-term stability with an Allan deviation below 10^−7^ Hz/Hz at 10 s averaging time. Meanwhile, microwave-to-optics conversion was achieved initially using a GaAs device based on an integrated, on-chip electro-optomechanical device that couples surface acoustic waves [147], driven by a resonant microwave signal to an optomechanical crystal featuring a 2.7 GHz mechanical mode. The mechanical mode in its quantum ground state was operated with minimal added thermal noise, while maintaining an optomechanical cooperativity >1, elucidating that microwave photons mapped into the mechanical resonator are effectively up-converted to the optical domain.

Bidirectional on-chip conversion between microwave and optical frequencies was later realized by piezoelectric actuation of this Gigahertz-frequency GaP optomechanical resonator. The large optomechanical coupling and the suppression of two-photon absorption in the material give rise to operating the device at optomechanical cooperativities greatly exceeding one [100]. Less than one thermal noise phonon was possibly reduced by using a pulsed up-conversion pump, where a high-impedance on-chip matching resonator was induced to mediate the mechanical load with the 50-Ω source.

A PhC cavity made of single-crystal, piezoelectric GaP integrated on prefabricated niobium circuits on an intrinsic silicon substrate [58] was exploited to exhibit spatially extended, sideband-resolved mechanical breathing modes at ∼3.2 GHz, with vacuum optomechanical coupling rates of up to *g*_0_/2π ≈ 300 kHz, which is illustrated in Figure 21(d). The mechanical modes are driven by integrated microwave electrodes via the inverse piezoelectric effect, so that the system was estimated to achieve an electromechanical coupling rate to a superconducting transmon qubit of ∼200 kHz. The optomechanical performance of GaP devices is summarized in Table 11.

**Table 10:** Third-order nonlinear effects using GaP devices.

Wavelength range	Structure	GaP fabrication technique	Cavity Q	Mechanism	Input power	Conversion efficiency	Result	Year	Reference
450 nm–600 nm	Bulk GaP			Supercontinuum	0–3.2 W		Visible light generation is a result of 520 nm pumped parametric gain, which amplifies weak SPM sidebands while simultaneously generating long-wavelength idler light	2020	[69]
950 nm–1,250 nm									
3.95 µm	Film	MOCVD		HHG			Max. pump 80 GW cm^2^	2021	[57]
1,650 nm	Film	Epitaxially grown GaP thin films		SHG/THG			3 × 10^−19^ m^2^ V^−2^ (third-order nonlinear susceptibility)	2023	[8]
1,557 nm	Microdisks	GaP (250 nm)/AlGaP (750 nm)/GaP epitaxially grown	6 × 10^4^	SHG/THG	8.7 mW		*η*_SHG_ = 1.1 × 10^−1^ % MW^−1^	2022	[82]
							*η*_DTHG_ = 3.2 × 10^−5^ % MW^−2^		
							*η*_CSFG_ = 3.2 × 10^−3^ % mW^−2^		
							ζ = 7.7 % mW^−1^		
400 nm–1,200 nm	Nanodisk	Sims.		SHG/DFWM			*η*_DFWM_ = 5.2 × 10^−3^ W^−^2	2021	[115]
1,539 nm	Ring resonator	Wafer bonding	2 × 10^4^	SHG/THG	3.7 mW			2018	[60]
1,545 nm–1,547 nm	Waveguide	MOCVD		FWM	14 mW	−3 dB		2018	[103]
1,560 nm–1,568 nm	Waveguide	MOCVD		FWM	10–20 dBm		3.5 × 10^−18^ W^−1^ m^2^ (material nonlinearity)	2019	[107]
1,450 nm–1,650 nm	Waveguide	GaP(300 nm)/Al_0.2_Ga_0.8_P/GaP(100) MOCVD	1.2 × 10^6^	Kerr comb generation	78.6 mW		3.57 GHz (max. estimated detuning)	2023	[24]
1,530 nm–1,565 nm (C-band)	Waveguide	Direct wafer bonding	>10^5^	Kerr comb generation	3 mW		Broadband >100 nm with comb spacing ranging from 100 to 250 GHz	2020	[16]
1,550 nm	Waveguide	Direct bonding process		Supercontinuum			Nonlinear parameter is 90.9 m^−1^ W^−1^ nonlinear refractive index 1.9 × 10^−17^ m^2^ W^−1^ propagation loss of 23.5 dB/cm	2023	[61]
1,550 nm	Waveguides	Micro-transfer printing		Supercontinuum	<100 W		Waveguide loss 6 dB/cm	2023	[22]

**Table 11:** Optomechanical and mechano-optical GaP devices.

Optical	Mechanical	Intrinsic	Loaded	Coupling	Optical	Mechanical	Cooperativity	Power	Year	Reference
wavelength	frequency	Q	Q	rate *g*_0_/2π	damping *κ*/2π	damping *γ*_m_/2π	*C*_0_ = 4*g*_0_^2^/*κγ*_m_			
1,520–1,625 nm	488 MHz	2.8 × 10^5^	N.A.	30 kHz (sim.)	0.668 GHz	763 kHz	5.3 × 10^−6^	2.1 mW	2014	[81]
1,538.98 nm	2.905 GHz	1.49 × 10^5^	3.79 × 10^4^	845 kHz	5.14 GHz	13.8 kHz	^a^4.03 × 10^−2^	25 nW/750 nW	2019	[99]
1,525.66 nm	3.280 GHz (A)	1.09 × 10^5^	6.73 × 10^4^	193 kHz (A)	2.92 GHz	^a^2.55 mHz (A)	^a^2.00 × 10^−5^ (A)	∼130 nW	2022	[58]
	3.314 GHz (B)			285 kHz (B)		^a^2.81 mHz (B)	^a^3.96 × 10^−5^ (B)			
	3.328 GHz (C)			294 kHz (C)		^a^2.56 mHz (C)	^a^4.62 × 10^−5^ (C)			
1,536 nm (with electro)	3.35 GHz	N.A.	5.55 × 10^4^	76 kHz	^a^3.52 GHz	^a^8.03 mHz	^a^8.17 × 10^−7^	224 μW – 1.1 mW	2023	[62]
1,493 nm	2.902 GHz	1.11 × 10^5^	N.A.	400 kHz	3.15 GHz	3.12 mHz	6 × 10^−5^	∼20 μW	2019	[59]
1,555.4 nm	2.799 GHz	N.A.	^a^4.6 × 10^4^	700 kHz	4.17 GHz	67 kHz	7.0 × 10^−3^	0.5 μW	2022	[100]
				(2.799 GHz)		(2.799 GHz)	(2.779 GHz)			
	2.790 GHz			272 kHz		191 kHz	3.7 × 10^−4^			
				(2.790 GHz)		(2.790 GHz)	(2.790 GHz)			

^a^Calculated from the data in the references.

Besides, nonclassical mechanical states guided in a suspended silicon phononic cavity-waveguide [148] were observed where multiple round trips of phonons between the source and the reflector. The long mechanical lifetime of almost 100 μs demonstrates the possibility of nearly lossless transmission of single phonons over, in principle, tens of centimeters.

More recently, an integrated transducer based on a planar superconducting resonator coupled to a silicon photonic cavity through a mechanical oscillator made of lithium niobate (320-nm-thick X-cut) on silicon [149] was demonstrated with a transduction efficiency of 0.9 % with 1 μW of continuous optical power and a spectral bandwidth of 14.8 MHz. At a repetition rate of up to 100 kHz, the added noise is limited to a few photons with short optical pulses, laying the foundations for distributed quantum computing.

## 5 Summary and outlook

Nanodevices have been extensively demonstrated in the GaP material system, covering a wide range of applications. The challenges of GaP are twofold: first, scattering loss hinders high-Q resonant cavities, with key problems including lattice mismatches, defects, and surface roughness [150]; second, lacking of standardized fabrication platform and design tools will limit its applications and slow the innovation progress [151], [152]. In light of this, we can anticipate a surge in the application of previously underrated GaP nanodevices in the near future, especially in the following areas.

### 5.1 Biological applications

In both 700–900 nm NIR-I and 1,000–1,700 nm NIR-II windows [153], GaP offers nearly lossless features along with some of the best light confinement capabilities. Additionally, GaP permits the transmission of light from 550 nm (indirect bandgap of 2.26 eV), covering most green and red fluorescence processes in optical imaging. Higher spatial resolution can be expected at GaP surfaces for optical imaging. Once optical losses become slightly more tolerable, the transmission of blue light above 450 nm (the direct bandgap of GaP) will enable its broader application in systems compatible with human vision.

### 5.2 Quantum applications

Entangled photon pairs become critical for emerging quantum technologies. Both single emitters and SPDC are transitioning from the near-infrared to the visible regime, facilitated by the availability of inexpensive and high-performance photodetectors. Therefore, GaP is increasingly considered one of the best candidates. On one hand, single emitters such as nitrogen vacancy centers [102], [106], [112], silicon vacancy centers [104], 2D WSe_2_ [85], 2D WS_2_ [154], and CdSe nanoparticles [79] were demonstrated on GaP nanodevices. On the other hand, the SPDC processes [55], [56], [80] using *d*_14_ can be distinct from the conventional *d*_33_ channels.

### 5.3 Telecommunications

Telecommunication and data communications in the data centers have been the main driving force for nanophotonics. Lossless GaP can be used to create photonic integrated circuits (PICs) that are highly efficient in light emission and detection, leading to smaller, more energy-efficient components for fiber optic networks and wireless communication systems, offering potentially higher speeds, higher-bandwidth, tunability, and lower power consumption than current technologies.

### 5.4 LiDAR

LiDAR (Light Detection and Ranging) systems have strong demands on light manipulations, i.e., precise beam steering [155], [156] as well as rapid modulations [157], [158]. This has implications that GaP can be a strong candidate in various fields where accurate and fast distance measurement is critical, such as autonomous vehicles and robotics.

### 5.5 Photonic computing

Photon-based computing, as opposed to traditional electron-based methods, has become critically important due to its higher speed, greater bandwidth, capability for multiple data streams, and importantly, its energy efficiency [159], [160]. The high speed of photons can lead to lower latency in data transmission, which is crucial for applications requiring real-time processing. GaP could be a game changer for its matched lattice with silicon, which offers negligible loss in both linear and two-photon absorptions, further reducing the power consumption for integrated devices. To achieve these goals, most of the nonlinear optical effects will be heavily involved. However, we note that there are still areas and effects awaiting experimental demonstration.

### 5.6 *χ* ^(2)^-based optical parametric generation, amplification, and oscillation

Although OPO using *χ* ^(3)^ nonlinear effects have been demonstrated on GaP nanodevices, the concept of *χ* ^(2)^-based optical parametric generation, amplification, and oscillation, such as optical parametric generation (OPG) – where a high-intensity pump photon is split into a signal photon and an idler photon – is yet to be fully explored. Second, optical parametric amplification (OPA) is similar to OPG but starts with an existing weak signal. The high-intensity pump beam amplifies this signal while also generating the idler beam. Once demonstrated by GaP, this process could level up the amplification of light with very short pulse durations and at wavelengths where conventional laser materials are inefficient or unavailable, which is essential for ultrafast pulse generation. Third, an OPO involves placing an OPG or OPA inside an optical cavity resonant for the signal and/or idler waves. *χ* ^(2)^-based GaP OPOs could be used to create tunable laser sources over a wide range of wavelengths, especially in the infrared spectrum, which are valuable in spectroscopy and other scientific applications for precision and control. These methods are also important in photonics research and emerging quantum technology applications, where control of photon properties is essential.

### 5.7 Optical modulator based on Pockels effect

The Pockels effect is a nonlinear optical phenomenon where the refractive index changes in response to an applied electric field. This effect is a cornerstone in the field of electro-optics and has significant applications in modulating and switching light. However, it seems missing in terms of using GaP. This could be due to the specific *r*_14,_ where the electric field has to be placed perpendicular to the polarization of light. This, in principle, should be unleashed at the nanoscale. The implementation of the Pockels effect can leverage the high-speed switches and electro-optic modulators, where the phase, polarization, or amplitude of light is controlled by an electric field.

### 5.8 Spontaneous four-wave mixing (SFWM)

In quantum state engineering, spontaneous four-wave mixing (SFWM), an alternative approach in quantum photonic technologies [161], [162], [163], has not yet been demonstrated on GaP devices. Two pump photons spontaneously decay into a photon pair, also requiring momentum conservation for the participating photons, which strongly limits the versatility of the resulting quantum states. We could foresee GaP nanodevices could underpin the relaxation of this constraint combined with resonances, expanding the new possibilities.

### 5.9 Brillouin scattering

Brillouin scattering as the interaction of light with acoustic phonons (sound waves) has not been explicitly investigated in GaP nanodevices. Brillouin lasers have been demonstrated on suspended silicon photonic–phononic waveguides [164]. On the other hand, many implementations rely on complicated fabrication schemes using nonstandard materials such as As_2_S_3_. Advanced stimulated Brillouin scattering has also been verified in multilayer silicon nitride waveguides as a microwave photonic notch filter with high rejection (30 dB) [165]. A recent renaissance in Brillouin scattering research has been driven by the increasing maturity of photonic integration platforms and nanophotonics, paving the way toward a new breed of chip-based devices that exploit acousto-optic interactions to create lasers, amplifiers, filters, delay lines, and isolators [166].

### 5.10 Optothermal and thermo-optical devices

The thermal-related properties of GaP have rarely been explored. These devices leverage the interaction between the optical and thermal properties within GaP, which is enhanced by its optical absorption. For example, nontoxic GaP nanoparticles may find applications for targeted cancer therapy, where they are heated using infrared light to kill cancer cells. On the other hand, it is not sure whether GaP’s efficient light absorption could enhance heat generation for thermoelectric generators.

Thermo-optical devices exploit the change in optical properties (like refractive index) of materials in response to temperature changes. Thermal-controlled GaP devices alter the refractive index upon temperature, making it suitable for optical switches and modulators.

This brief review of GaP emphasizes the details of fabrication and summarizes all types of existing devices on nonlinear optical effects. Besides the demonstrated applications and the undemonstrated effects, we believe with the rapid underpinning of the fundamental operation of GaP, it is flourishing in microwave photonics, attosecond science, and terahertz technology and concludes with perspective for future directions.
